# Nucleocytoplasmic transport senses mechanical forces independently of cell density in cell monolayers

**DOI:** 10.1242/jcs.262363

**Published:** 2024-09-09

**Authors:** Ignasi Granero-Moya, Valeria Venturini, Guillaume Belthier, Bart Groenen, Marc Molina-Jordán, Miguel González-Martín, Xavier Trepat, Jacco van Rheenen, Ion Andreu, Pere Roca-Cusachs

**Affiliations:** ^1^ Institute for Bioengineering of Catalonia (IBEC), Barcelona Institute of Science and Technology (BIST), 08014 Barcelona, Spain; ^2^ University of Barcelona, 08036 Barcelona, Spain; ^3^ Oncode Institute, 1066 CX Amsterdam, The Netherlands; ^4^ Department of Molecular Pathology, The Netherlands Cancer Institute, 1066 CX Amsterdam, The Netherlands; ^5^ Eindhoven University of Technology, Department of Biomedical Engineering, PO Box 513, 5600 MB Eindhoven, The Netherlands; ^6^ Institució Catalana de Recerca i Estudis Avançats (ICREA), 08010 Barcelona, Spain; ^7^ Centro de Investigación Biomédica en Red en Bioingeniería, Biomateriales y Nanomedicina (CIBER-BBN), 08014 Barcelona, Spain; ^8^ Biofisika Institute (CSIC, UPV/EHU), 48940 Leioa, Spain; ^9^ Ikerbasque, Basque Foundation for Science, 48009 Bilbao, Spain

**Keywords:** Mechanotransduction, Sensor, Mechanobiology, Cell nucleus

## Abstract

Cells sense and respond to mechanical forces through mechanotransduction, which regulates processes in health and disease. In single adhesive cells, mechanotransduction involves the transmission of force from the extracellular matrix to the cell nucleus, where it affects nucleocytoplasmic transport (NCT) and the subsequent nuclear localization of transcriptional regulators, such as YAP (also known as YAP1). However, if and how NCT is mechanosensitive in multicellular systems is unclear. Here, we characterize and use a fluorescent sensor of nucleocytoplasmic transport (Sencyt) and demonstrate that NCT responds to mechanical forces but not cell density in cell monolayers. Using monolayers of both epithelial and mesenchymal phenotype, we show that NCT is altered in response both to osmotic shocks and to the inhibition of cell contractility. Furthermore, NCT correlates with the degree of nuclear deformation measured through nuclear solidity, a shape parameter related to nuclear envelope tension. In contrast, YAP is sensitive to cell density, showing that the YAP response to cell–cell contacts is not via a mere mechanical effect of NCT. Our results demonstrate the generality of the mechanical regulation of NCT.

## Teaser

**Highlighted Article:** The fluorescent sensor of nucleocytoplasmic transport, Sencyt, is used to show that nucleocytoplasmic transport in cell monolayers responds to mechanical changes in the nucleus but not to cell density, unlike YAP.

## INTRODUCTION

Cells sense and respond to their mechanical context in a process called mechanotransduction. Mechanotransduction is essential in physiological situations, such as organ development (Hamant and Saunders, 2020) and embryogenesis (Brunet et al., 2013), and also in pathological settings, for instance tumor progression (Broders-Bondon et al., 2018). One of the cell elements involved in mechanotransduction is the cell nucleus, which responds to both intracellular and extracellular forces through several mechanisms. These mechanisms involve changes in chromatin architecture (Nava et al., 2020), in the conformation and localization of nucleoskeletal elements, such as lamins (Philip and Dahl, 2008; Swift et al., 2013), in nuclear membrane tension (Lomakin et al., 2020; Venturini et al., 2020), and in the localization and activity of transcriptional regulators (Elosegui-Artola et al., 2017; Tajik et al., 2016). In single adhesive cells, transcriptional regulators including YAP (also known as YAP1) (Elosegui-Artola et al., 2017), Twist and Snail family proteins, and SMAD3 (Andreu et al., 2022) localize to the nucleus in response to force due to changes in nucleocytoplasmic transport (NCT). Specifically, force applied from the extracellular matrix to the nucleus by actomyosin contractility increases nuclear membrane tension, nuclear pore complex (NPC) diameter and diffusion through NPCs (Elosegui-Artola et al., 2017; Schuller et al., 2021; Zimmerli et al., 2021). Both passive and facilitated diffusion (i.e. passive and active transport) are affected by force, but to different extents. This causes a differential effect that leads to force-dependent nuclear or cytoplasmic accumulation of proteins depending on the balance between their passive transport properties and their active transport properties (governed by their nuclear localization or export sequences) (Andreu et al., 2022).

**Fig. 1.**
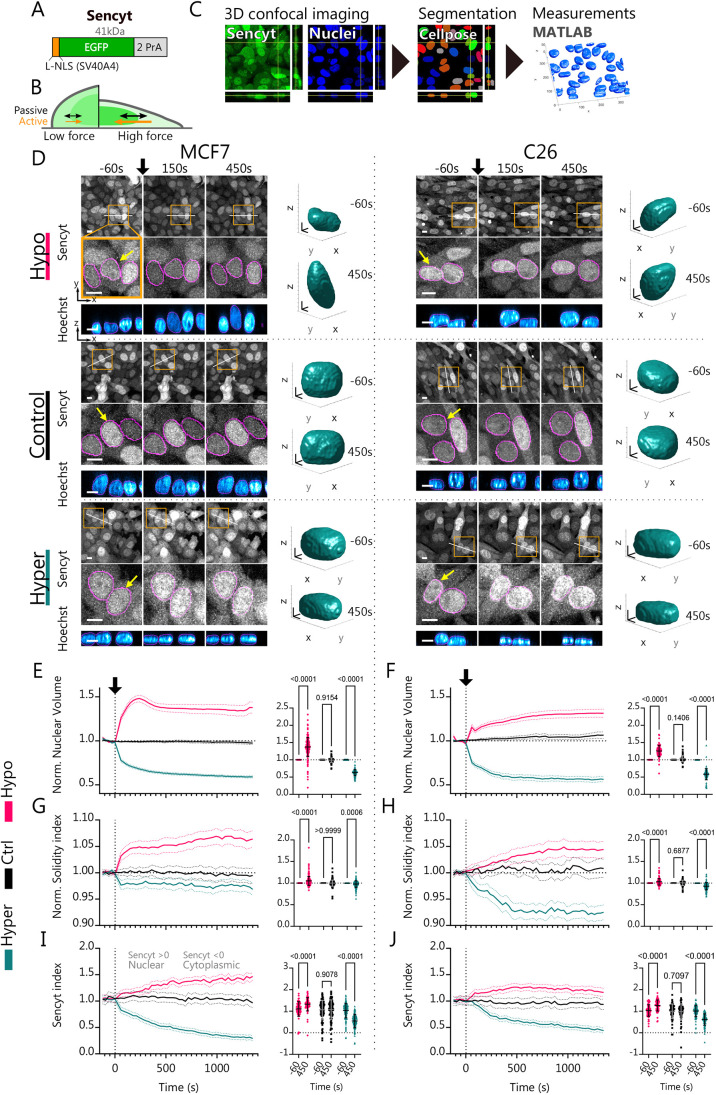
**Hypo- and hyper-osmotic shocks increase and decrease nucleocytoplasmic transport respectively.** (A) Schematic representation of Sencyt. Its elements include: (i) an NLS (SV40A4) based on that from the SV40 virus, but with a point mutation to reduce affinity to importins; (ii) an EGFP molecule for visualization; and (iii) two repeats of the inert protein PrA to confer a final molecular mass of 41 kDa, thereby regulating passive diffusion. (B) Scheme of the Sencyt response to force. When force exerted to the nucleus increases, passive diffusion increases, but active transport increases even more. This increases Sencyt nuclear localization (Andreu et al., 2022). (C) Image processing workflow chart. Cells stably transfected with Sencyt are imaged for Sencyt and nuclei (Hoechst 33342 label). Nuclei are then segmented, and nuclei shape parameters and Sencyt index are calculated (see Materials and Methods). (D) Representative images as a function of time for cells submitted to hypo-, control or hyper-osmotic shocks, both for MCF7 and C26 cell lines. The black arrow indicates the beginning of treatment. In the top panels, orange squares indicate areas that are magnified in middle panel, white lines indicate the location of vertical nuclear cross-sections shown in bottom panel. Magenta lines show nuclear mask limits. Note that due to changes in cell volume, hypo- and hyper-osmotic shocks decrease and increase overall fluorescence intensity levels, respectively. Right, 3D nuclei mask renders of indicated nuclei (yellow arrow), at indicated timepoints. Scale bars: 10 µm (2D images), 4 µm (3D nuclei renders). (E,F) Nuclear volume measurements normalized to the first five timepoints, and statistics pre- and post-treatment (*n*=170, 130, 188, 81, 72 and 107 cells). (G,H) Solidity index measurements normalized to the first five timepoints and statistics pre- and post-treatment (*n*=230, 170, 231, 121, 104 and 142 cells). (I,J) Sencyt index measurements and statistics pre- and post-treatment (*n*=66, 101, 152, 60, 63 and 103 cells). Sencyt index is defined as the logarithm in base 2 of the ratio of the mean nuclear fluorescence and the mean cytoplasmic fluorescence (see Materials and Methods). *P*-values calculated with two-way ANOVA corrected with Šídák's multiple comparisons test. Error bars represent 95% c.i. for timelapse graphs and s.d. for statistical graphs. All data are from three independent repeats.

The role of NCT in mechanotransduction is thus established for single cells, but if and how it applies to multicellular systems is unclear. In multicellular systems, cell mechanotransduction involves a complex interplay between cell–matrix and cell–cell adhesion (Aragona et al., 2013; Maniotis et al., 1997). Furthermore, cell–cell adhesion per se also regulates transcriptional regulators, such as YAP, in ways that could be independent of mechanotransduction mechanisms or NCT (Aragona et al., 2013; Zhao et al., 2007, 2008). Thus, to what extent NCT changes can explain mechanotransduction responses in multicellular systems is unknown. To address this, we need a NCT reporter that is sensitive to mechanical forces, but not to signaling pathways (such as the Hippo pathway that regulates YAP). In our previous work (Andreu et al., 2022), we screened a battery of synthetic constructs that expressed inert, freely diffusing proteins that only interact with the active transport machinery through nuclear localization sequences (NLSs). These proteins showed different facilitated and passive transport rates, and some of them had mechanosensitive shuttling rates and localization. In single fibroblasts, the synthetic protein L_NLS-41 kDa (Fig. 1A) presented the biggest mechanosensitivity, defined as the change in localization in response to force. Indeed, in response to force applied to the nucleus, L_NLS-41 kDa showed increased rates of both passive and active nuclear transport (Fig. 1B). However, active transport was more affected by force, leading to a force-dependent accumulation in the nucleus.

Owing to these properties, L_NLS-41 kDa is an appropriate mechanosensitive NCT reporter, which, for convenience, we have renamed as SEnsor of NucleoCYtoplasmic Transport (Sencyt). In this work, we use Sencyt in cell monolayers, and show that NCT responds to mechanical inputs but not cell–cell contacts, thereby separating the two types of inputs that are the major regulators of YAP.

## RESULTS

To evaluate the role of NCT in the mechanotransduction of multicellular systems, we used two different cell lines stably expressing Sencyt – MCF7 and C26. Both are cancer cell lines, but present different characteristics. MCF-7 are epithelial cells isolated from metastatic adenocarcinoma of a human breast tumor and are used for breast cancer research and many mechanobiological studies. They have an epithelial phenotype (Ahlstrom and Erickson, 2007), with strong cell–cell adhesions. C-26 is a murine colon adenocarcinoma cell line (also named MCA-26, CT-26 and Colo-26) (Corbett et al., 1975). It has a more mesenchymal phenotype, presenting thus an interesting contrast to MCF7. Confirming these phenotypes, MCF7 cells exhibited clear cell–cell adhesions containing E-cadherin, whereas C26 cells did not (Fig. S1A–D). MCF7 cells, but not C26 cells, also showed a clear apico-basal polarity as assessed through their actin cytoskeleton (Fig. S1E). To test the mechanosensitivity of NCT in these two cell lines in a multicellular context, we carried out two types of mechanical perturbations – osmotic shocks and inhibition of the forces exerted by the cell actin cytoskeleton.

### Hypo- and hyper-osmotic shocks increase and decrease nucleocytoplasmic transport respectively

**Fig. 2.**
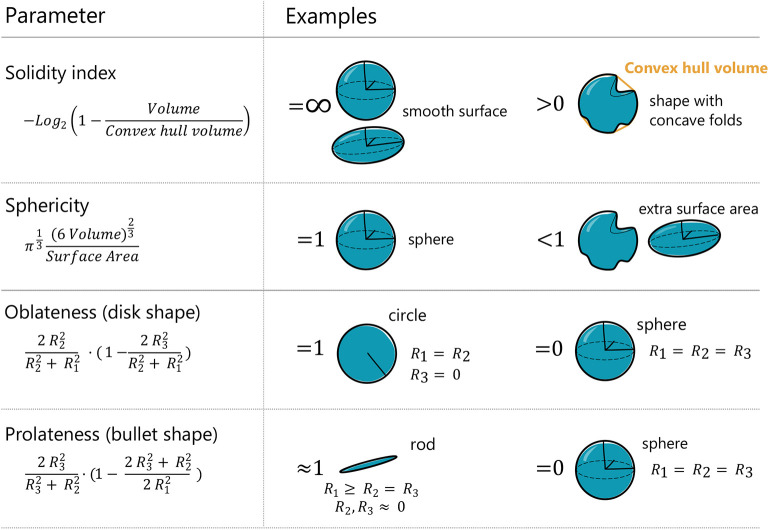
**Nuclear shape parameters description.** *R*_1_, *R*_2_ and *R*_3_ correspond to the three radii (from the largest to the smallest; *R*_1_≥*R*_2_≥*R*_3_) of an ellipsoid fitted to the segmented nucleus. Volume and surface areas are the measured volume and surface area of the nucleus as obtained from nuclear segmentation. Convex hull volume is the volume of the convex hull, defined as the smallest convex shape (that is, not containing any concave folds) that encloses the nucleus.

Osmotic shocks have been widely used to alter mechanical conditions for the nucleus (Schuller et al., 2021; Venturini et al., 2020; Zimmerli et al., 2021). In this work, we have used the osmotic stress conditions previously used in similar works (Elosegui-Artola et al., 2017) to induce nuclear swelling or shrinking, thereby affecting the tension in the nuclear envelope (Dahl et al., 2004; Enyedi et al., 2016), which in turn affects NPC diameter (Zimmerli et al., 2021). We applied the osmotic shocks on cells while we imaged confocally Sencyt and the nucleus (through Hoechst 33342 staining) (Fig. 1C). The well-described responses for hypo-osmotic shocks include an inflow of water into the cell that causes an increase of cell and nuclear volumes, and a decrease in the concentration of solutes inside of the cell (Churney, 1942; Finan et al., 2009; Lemière et al., 2022). Opposite to hypo-osmotic shocks, hyper-osmotic shocks cause an outflow of water from the cell, which causes a decrease in the cell and nuclear volumes and increase the concentration of solutes (Churney, 1942; Finan et al., 2009; Lemière et al., 2022). To track changes in nuclear volume, and nuclear shape in general, we segmented nuclear images in 3D, and calculated different shape parameters (see Materials and Methods and Fig. 2). By measuring volume changes, we reproduced these trends (Fig. 1D–F; Figs S2 and S3). For both cell lines, hypo-osmotic shocks increased nuclear volume (by a 50% for MCF7 and a 30% for C26). Inversely, hyper-osmotic shocks reduced nuclear volume (up to 40% in both cell lines, Fig. 1E,F; Figs S2 and S3A,B). In C26 cells, the hypo-osmotic shock changes are milder than in MCF7, potentially due to different properties of the nucleoskeleton or initial differences in cell and nuclear osmolarity, which resists nuclear deformations. In MCF7, we also observed an initial nuclear volume increase by 50%, followed by a decrease to 40%. This could be explained by there being an adaptative mechanism, by which cells decrease hypo-osmotic stress by reducing the internal ion concentration (Enyedi et al., 2016; Hoffmann et al., 2009; Lang et al., 1998).

The nucleus is delimited by a double lipidic membrane that is not elastic (Hallett et al., 1993; Needham and Nunn, 1990). Assuming it is finite, increasing the volume of a wrinkled nucleus should, first, increase the nuclear membrane area until the exhaustion of the membrane reservoirs, and second, decrease the number of wrinkles and make the nucleus smoother by increasing nuclear membrane tension, as previously suggested (Niethammer, 2021). Regarding the first part, nuclear surface area increased and decreased for both cell lines when submitted to hypo- and hyper-osmotic shocks, respectively (Fig. S3E,F). To tackle the second part, we measured the nuclear Solidity index (see Materials and Methods). The Solidity index quantifies the overall concavity of a 3D volume, with high values corresponding to a taut nucleus and low values corresponding to a wrinkled nucleus (Fig. 2). It can thus be understood as an indirect estimate (but not a direct measurement) of nuclear membrane tension, given that we would expect a high Solidity index for a taut nucleus submitted to high membrane tension. Consistent with this framework, the Solidity index increased in the hypo shock condition, and decreased in the hyper shock condition (Fig. 1G,H), suggesting changes in nuclear envelope tension.

Next, we measured the Sencyt index, defined as the logarithm (in base 2) of the nuclear-to-cytoplasmic ratio of Sencyt signal (see Materials and Methods). Thus, a positive Sencyt index indicates nuclear localization, a negative one indicates cytoplasmic localization, with zero being an equal distribution between both compartments. By measuring changes in the Sencyt index, we can track the ability of the cell NCT system to localize a cargo protein in the nucleus. Upon hypo-osmotic shocks, the Sencyt index increased along with nuclear volume and solidity (Fig. 1I,J). By contrast, upon hyper-osmotic shocks, the Sencyt index decreased along with nuclear volume and solidity (Fig. 1I,J). As a control, we transfected the cells with mCherry, which behaves in a completely diffusive way, and evenly occupies all accessible spaces in the cell. The localization of mCherry was not affected by osmotic shocks, except for a small increase in response to hyper-osmotic shocks in MCF7 cells (i.e. in the opposite direction to the effect on Sencyt; Fig. S3G–L). Thus, the changes in Sencyt index are not due to potential effects in the water fluxes, geometry, available space or the effective volume that Sencyt can occupy in the two compartments. Therefore, our data show that osmotic shocks regulate the ability of NCT to accumulate cargoes in the nucleus, in a manner consistent with a role for nuclear envelope tension.

### Myosin II and Arp2/3 inhibition decrease NCT

As a second mechanical perturbation, we inhibited actomyosin activity. To this end, we combined 25 µM para-NitroBlebbistatin, which inhibits myosin II by preventing its ATPase activity (Képiró et al., 2014), and 50 µM CK666, which binds to and inhibits Arp2/3, impairing actin branching and formation of lamellipodia (Beckham et al., 2014; Hetrick et al., 2013; Nolen et al., 2009). Of note, we combined both drugs because, in an epithelial context, para-NitroBlebbistatin alone is not sufficient to reduce nuclear mechanotransduction. Indeed, our previous work has shown that myosin contractility inhibition alone in epithelial cells can lead to increased cell spreading. This cell spreading induces nuclear deformation, increasing (rather than decreasing) nuclear mechanotransduction, as assessed via YAP nuclear concentration (Kechagia et al., 2023). The addition of CK666 prevents the increase in cell spreading by inhibiting lamellipodia formation, reducing YAP nuclear localization (Kechagia et al., 2023).

**Fig. 3.**
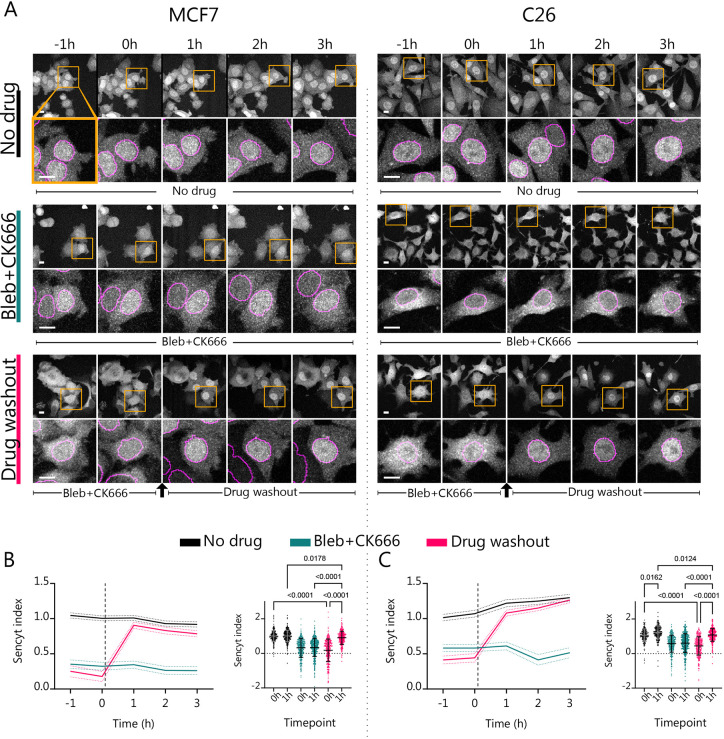
**Myosin II and Arp2/3 inhibition decreases NCT.** (A) Representative images as a function of time of cells submitted to different treatments, both for MCF7 and C26 cell lines. In top panels, yellow squares indicate areas that are magnified in the lower panel. Magenta lines show nuclear mask limits. Scale bars: 10 µm. (B,C) Corresponding measurements and statistics for the Sencyt index (*n*=301, 307, 376, 413, 327 and 299 cells). *P*-values calculated with Kruskal–Wallis test corrected with Dunn's multiple comparisons test. Error bars represent 95% c.i. for timelapse graphs and s.d. for statistical graphs. All data are from three independent repeats.

In this set-up, we performed three conditions in parallel: (1) a negative control treated with the vehicle, (2) a positive control treated with the drug combination, and (3) a drug washout condition. In the drug washout condition, the drugs were washed out after 2 h of imaging (Fig. 3A). Then, we analyzed the changes in Sencyt index with time in all three conditions. Treating the cells with the drug combination decreased the Sencyt index when compared with non-treated cells (Fig. 3B,C). In the case of the drug washout condition, the levels of NCT were mostly restored by 1 h after drug washout, although there were still residual effects (Fig. 3B,C). Surprisingly, when we checked for changes in nuclear volume and the Solidity index before and after the treatment there were no remarkable trends and no significant changes (Fig. S4A–D). Thus, mechanical force can affect NCT, even without clear nuclear deformations. To explain this, we hypothesize that there might be changes in nuclear envelope tension that do not require high deformations or measurable changes in solidity, as the nuclear envelope is a planar mechanical stiff material (Hallett et al., 1993; Needham and Nunn, 1990).

### Increasing cell spreading increases NCT

**Fig. 4.**
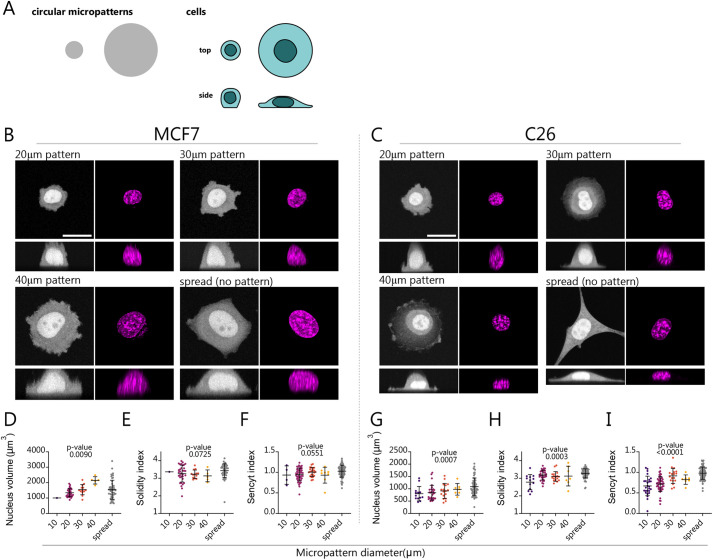
**Restricting cell spreading with micropatterns affects nuclear shape and decreases NCT.** (A) Cells were seeded on micropatterned circular islands of different diameter coated with fibronectin. (B,C) Representative images of MCF7 and C26 cells expressing Sencyt and stained with Hoechst 33342 cultured in circular fibronectin patterns of indicated diameter and on unpatterned substrates. Top rows show maximum projection images to visualize cell shape, bottom rows show *x-z* views (note that *x-z* views show fluorescence from individual confocal slices and thus best indicate Sencyt nuclear and cytoplasmic levels). Scale bars: 20 μm. (D–I) Nuclear volume, Solidity index and Sencyt index for MCF7 and C26 cells cultured in circular patterns of indicated diameter and on homogeneous fibronectin substrate. MCF7, *n*=4, 47, 22, 8 and 74 cells; C26, *n*=26, 49, 18, 7 and 91 cells per condition. Black lines represent mean±s.d. *P*-values are calculated using nonparametric Kruskal–Wallis test. All data are from three independent repeats.

To further verify the effect of mechanics on NCT, we applied a third type of mechanical perturbation, this time in the context of single cells. We seeded single cells on substrates coated with micropatterned circles of fibronectin of different sizes (Fig. 3A). In this way, cells spreading was constrained only to the patterns. It has previously been shown using micropatterns that increased cell spreading leads to increased force generation by cells (Roca-Cusachs et al., 2008; Tan et al., 2003), affecting in turn nuclear shape (Alisafaei et al., 2019; Versaevel et al., 2012). Indeed, for both cell types, increased cell spreading led to changes in nuclear shape, as indicated by increased nuclear volume (Fig. 4D,G). For C26 cells, spreading also led to a progressive increase in the sencyt index and in nuclear solidity (Fig. 4C,H,I). In contrast, spreading in MCF7 cells did not affect either the sencyt index or solidity (Fig. 4B,E,F). This lack of response of MCF cells could potentially be due to nuclear mechanoprotection mechanisms associated with epithelial cells (Kechagia et al., 2023). Thus, cell mechanics as controlled through cell spreading also affects NCT in a way that correlates with nuclear solidity.

### Sencyt correlates with nuclear shape but not cell density in monolayers

**Fig. 5.**
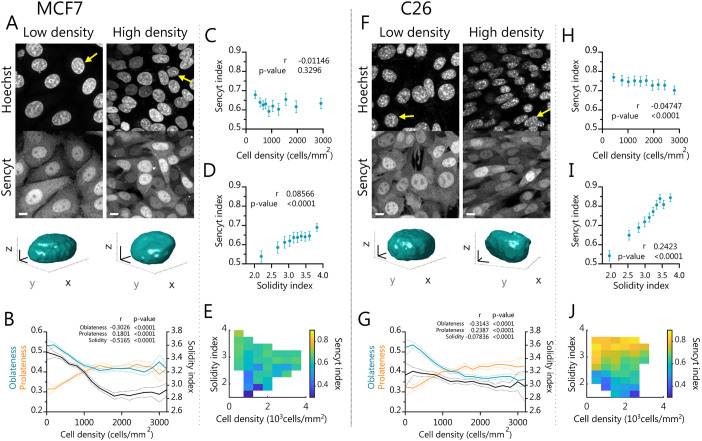
**Sencyt correlates better with nuclear shape than cell density in monolayers.** (A,F) Representative live images of cells in low and high density showing Sencyt and nucleus staining. Bottom, 3D rendering of example nuclei (indicated with a yellow arrow). Scale bars: 10 µm (2D images); 4 µm (3D renders). (B,G) Nuclear shape parameters versus density (*n*=20560 and 16711 cells). (C,H) Sencyt index versus cell density (*n*=7237 and 7522 cells). (D,I) Sencyt index versus Solidity index (*n*=7865 and 7647 cells). (E,J) Average Sencyt index as a function of Solidity index and cell density, for both cell types (*n*=7118 and 7522 cells). *P*-values calculated with a two-tailed non-parametric Spearman correlation test. Error shading and error bars represent 95% c.i. A–E show results for MCF7 cells and F–J for C26 cells. All data include are from three independent repeats.

To better understand the role of nuclear shape, we studied its relationship with Sencyt in cell layers without imposed mechanical perturbations. To this end, we seeded cell monolayers laterally confined by a polydimethylsiloxane (PDMS) gasket. After removing the gasket, cells spread for 24 h, leading to monolayer areas with very different cell densities (Fig. 5A,F). Performing the same image analyses as in the previous experiments, we observed that the different cell densities also led to different nuclear shapes (Fig. 5A,B,G; Fig. S2). Specifically, decreased density, corresponding to increased cell spreading, led to progressive deformation of the nucleus, as indicated by increased oblateness and decreased prolateness (Fig. 5B,G; see parameter description in Fig. 2). As expected, this also led to an increase in the Solidity index.

Next, we analyzed how the Sencyt index correlated with cell density and with the Solidity index. The Sencyt index did not correlate with cell density for MCF7 cells (Fig. 5C) and correlated only very mildly for C26 cells (Fig. 5H). In contrast, the Sencyt index correlated with the Solidity index in both cell lines, with a higher correlation for C26 and milder one for MCF7 cells (Fig. 5D,I). In summary, the Sencyt index correlated much more with the Solidity index than with cell density for both cell lines (Fig. 5E,J). In fact, solidity was the nuclear geometrical parameter that best correlated with the Sencyt index for both cell types (Fig. S5). Interestingly, C26 cells exhibited higher correlations between Sencyt and overall nuclear shape parameters. Differences between cell lines could arise from several factors, including different nuclear mechanical properties, which depend on cell type (Hobson et al., 2020; Kechagia et al., 2023). This might lead to different tension–shape relationships.

### Cell layers show different regulation for Sencyt and for YAP

**Fig. 6.**
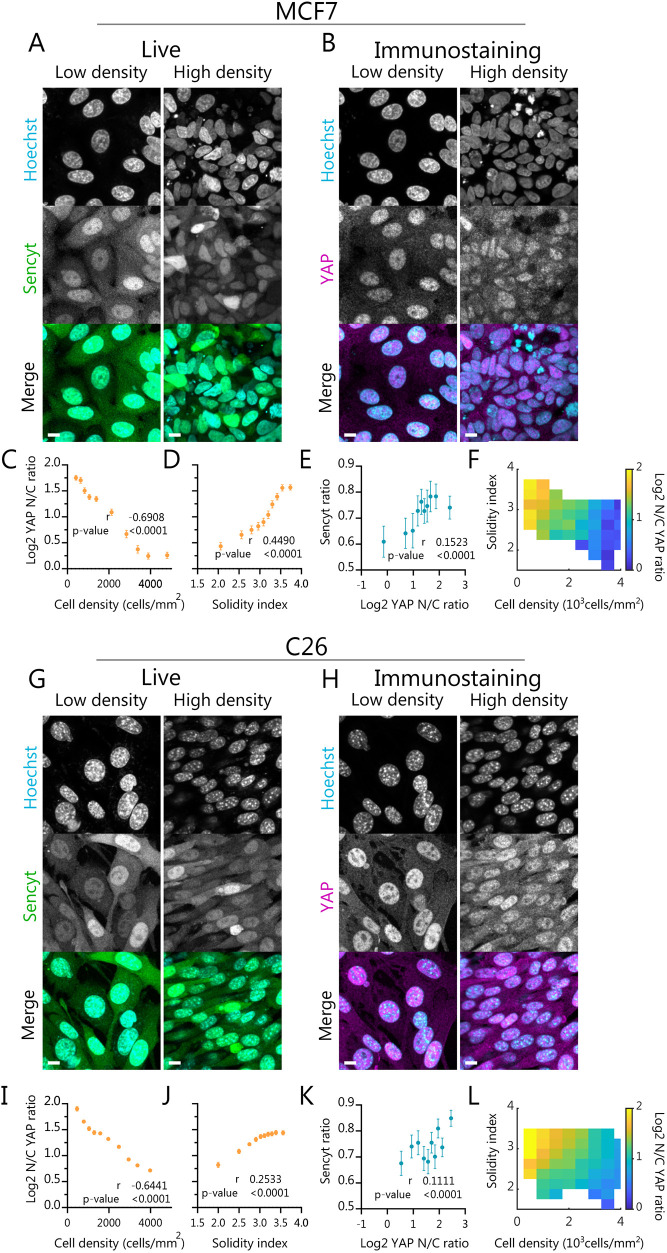
**Cell layers show different regulation for Sencyt and for YAP.** (A,G) Representative images of cells in low and high density showing Sencyt and nuclei staining. (B,H) Representative images of the fixed same cells in low and high density for YAP immunostaining and nuclei staining. Scale bars: 10 µm. (C,I) Log2 nuclear-to-cytoplasmic (N/C) YAP ratio versus cell density (*n*=4889 and 7204 cells). (D,J) Log2 N/C YAP ratio versus Solidity index (*n*=4889 and 7204 cells), (E,K) Cell-by-cell correlation of Sencyt index versus Log2 N/C YAP ratio (*n*=1548 and 1782 cells). (F,L) Log2 N/C YAP ratio in color versus cell density and Solidity index, for MCF7 and C26 cell lines, respectively (*n*=4679 and 7050 cells). *P*-values calculated with two-tailed non-parametric Spearman correlation test. Error bars represent 95% c.i. A–F show results for MCF7 cells and G–L for C26 cells. All data include are from three independent repeats.

Finally, we set out to understand whether NCT in monolayers is affected in the same way as YAP, a well-known transcription factor that has NCT-regulated mechanosensitivity (Elosegui-Artola et al., 2017), but which also undergoes complex biochemical regulation through the Hippo pathway (Piccolo et al., 2014). To this end, we immunostained for YAP in the same cell samples we imaged live for Sencyt (Fig. 6A,B,G,H). The YAP nuclear-to-cytoplasmic concentration ratio strongly correlated with density in both cell lines (Fig. 6C,I). This is an expected behavior given that YAP nuclear localization has been proven to depend on cell–cell contacts. Indeed, an increase in the number of cell–cell contacts decreases nuclear localization, decreasing proliferation (Aragona et al., 2013; Dupont et al., 2011; Zhao et al., 2007, 2008).

The YAP nuclear-to-cytoplasmic ratio also correlated with solidity in both cell lines (Fig. 6D,J), although to a lesser degree than with cell density. Overall, the combined effects of both factors were clearly visible (Fig. 6F,L). Correlations with solidity were similar for YAP and Sencyt in C26 cells (Figs 5L and 6J), but much higher for YAP in MCF7 cells (Figs 5D and 4D). Furthermore, for both cell lines, Sencyt and YAP localization correlated significantly with each other, more so in MCF-7 than in C-26 cells (Fig. 6E,K). However, correlations were significant but low, likely reflecting the fact that both parameters are not molecularly tied, and that the different layers of YAP regulation reduce the correlations. In fact, YAP localization correlated better than Sencyt index with most nuclear shape and other geometrical parameters (Fig. S5). YAP also followed the same trends than sencyt in response to cell spreading in micropatterns, showing changes in C26 but not MCF7 cells (Fig. S6).

## DISCUSSION

In this work, we harness the Sencyt sensor to demonstrate that NCT in cell monolayers is regulated by mechanical stimuli, leading to altered nuclear accumulation of shuttling proteins. The role of mechanics in NCT had been previously demonstrated in single cells, in response to either increased substrate stiffness, force applied to the nucleus (Andreu et al., 2022; Elosegui-Artola et al., 2017) or hypo-osmotic shocks (Zimmerli et al., 2021). Here, we demonstrate that mechanics also plays a role in multicellular systems in response to both hypo and hyper-osmotic shocks and to cell contractility. The mechanism involved is likely the same – increased nuclear membrane tension caused by applied force (Dahl et al., 2004; Enyedi et al., 2016), a subsequent increase in the diameter of the NPC (Zimmerli et al., 2021) and resulting differential alteration in passive versus facilitated diffusion through NPCs (Andreu et al., 2022).

Certainly, other factors beyond nuclear envelope tension could also be playing a role. Hyper-osmotic shocks have been shown to slow intracellular signaling due to molecular crowding (Miermont et al., 2013), and to decrease nuclear import (Ng et al., 2014) by impairing the Ran system (Kelley and Paschal, 2007). Furthermore, osmotic swelling due to tissue damage, and subsequently increased nuclear envelope tension, induces signaling by translocating cytosolic phospholipase A2 (cPLA2) and 5-lipoxygenase (5-LOX) to the inner nuclear membrane (Cho and Stahelin, 2005; Enyedi et al., 2016). The contribution of these different mechanisms in response to specific perturbations remains to be elucidated. However, the common response to very different stimuli (osmotic shocks, contractility inhibition and cell spreading), combined with the correlation with nuclear solidity, strongly suggest a role for nuclear envelope tension and direct effects on NPC permeability.

Comparing some of our results leads to interesting implications. First, mechanically induced changes in NCT can occur both with nuclear deformation (in response to osmotic shocks and cell adhesion) and without (in response to contractility inhibition). This suggests that mechanical perturbations can affect NCT and likely nuclear envelope tension without visible changes in nuclear shape. This could potentially be explained by different means of transmitting force – through global nuclear swelling in the case of osmotic shocks, versus specifically through the linker of nucleoskeleton and cytoskeleton (LINC) complex in response to cell contractility. Indeed, in our previous work we found a very different response of Sencyt in single cells when nuclei were deformed in the presence or absence of LINC complexes (Andreu et al., 2022). Such differences could also explain the fact that nuclear solidity and the Sencyt index correlate, but with rather low correlation values. Still, larger deformations will lead to larger effects, as indicated by the correlations between Sencyt index and Solidity index.

Second, we found very interesting differences when comparing the Sencyt versus YAP responses. Importantly, both Sencyt and YAP responded to nuclear solidity, but only YAP showed a clear response to cell density. This differential behavior allowed us to decouple the effects of mechanics and cell–cell adhesion, showing that the role of cell–cell adhesion in YAP modulation cannot be explained by mechanical effects on NCT. This is likely due to the several layers of YAP regulation, and specifically the role of cell–cell adhesion in the Hippo pathway (Aragona et al., 2013; Zhao et al., 2007, 2008). Interestingly, even if it also responded to density, YAP nuclear localization correlated better than Sencyt with nuclear solidity, at least for MCF7 cells. This suggests that the properties of YAP might have evolved to result in a more optimal mechanosensor than the synthetic Sencyt. In the future, further optimization of Sencyt might increase its sensitivity, potentially revealing interesting insights on how physiological mechanosensitive molecules such as YAP evolved.

Finally, there are also interesting differences between MCF7 cells (with epithelial phenotype) and C26 cells (with mesenchymal phenotype). Correlations between nuclear shape (solidity) and Sencyt are better for C26 than MCF7 (Fig. 5), and MCF7 cells do not even alter nuclear solidity, Sencyt or YAP in response to cell spreading (Fig. 4; Fig. S6). This suggests a higher mechanosensitivity of the nucleus in C26 cells, consistent with the known robust mechanosensing properties of mesenchymal cells. In contrast, the range of YAP nuclear-to-cytoplasmic ratio values in response to cell density is slightly higher in MCF7 cells (Fig. 6), consistent with a more important role of cell–cell adhesion in epithelial cells.

Methodologically, our results also show that a sensor of nucleocytoplasmic transport (Sencyt), together with image analysis, is a valuable tool that can be used to understand NCT regulation in multicellular environments *in vitro* and potentially *in vivo*, merely by using confocal fluorescence in live imaging. Using Sencyt is likely to reveal much finer NCT regulation than merely employing fluorophores tagged with a strong NLS sequence, as those strongly localize to the nucleus unless NCT is acutely disrupted. Potentially, Sencyt might for instance be used to identify other mechanosensitive transcriptional regulators purely regulated by NCT and not cell–cell adhesion in multicellular systems, overriding YAP-like regulation systems. Beyond mechanics, it could also be used to study alterations in NCT due to other factors.

## MATERIALS AND METHODS

### Cell lines

MCF-7 and C-26 cells were cultured in Dulbecco's modified Eagle medium (DMEM; Gibco, 41966-029) supplemented with 10% heat-inactivated fetal bovine serum (Sigma-Aldrich, 9040-46-8), L-glutamine (2 mM; Gibco; 25030-024) and penicillin-streptomycin (100 U/ml; Gibco,15070-063) in a humidified atmosphere with 5% CO_2_ at 37°C in humidified atmosphere. C-26 was kindly provided by Onno Kranenburg (Utrecht University, The Netherlands) and MCF-7 cells were from the van Rheenen laboratory (Netherlands Cancer Institute, The Netherlands). Cell lines were checked periodically for mycoplasma infection and for their epithelial and mesenchymal nature through immunostaining of E-cadherin. A plasmid transiently expressing Sencyt was previously described as L_NLS 41 kDa (Andreu et al., 2022) and is available through Addgene (Addgene plasmid #201342; RRID: Addgene_201342). For the creation of stable cell lines expressing Sencyt, pLentiPGK coding for SV40A4-EGFP-2PrA (Andreu et al., 2022) was cloned using the primers to excise it from the parental plasmid (Infusion_SV40A4-EGFP-2PrA_Fwd, 5′-CGGTACCGCGGGCCCATGGGCCCAAAAAAGGC-3′ and Infusion_SV40A4-EGFP-2PrA_Rev, 5′-GAAAGCTGGGTCTAGACCACTTTGTACAAGAAAGCTGGGTCGG-3′) and the In-Fusion HD Cloning Plus kit (638911, Takara Bio) according to the manufacturer's recommendations. The plasmid was then used for viral production in HEK293T (ATCC^®^ CRL-1573™) of low passage in Iscove's modified Dulbecco's medium (IMDM; Gibco, 21980-032) supplemented with 10% heat-inactivated FBS (Sigma-Aldrich, 9040-46-8) and 1% penicillin-streptomycin (Gibco, 15070-063). Reagents used were: 2.5 M CaCl2, 0.1× TE buffer, 2× HBS pH 7.12 (made freshly as previously described; Dull et al., 1998). Cell lines were transduced with a mix of supernatant containing virus and polybrene (Sigma H9268 suspended at 4 mg/ml in sterile water, 1:1000), at 37°C for 24 h. Transduced cells were selected by Hygromycin B Gold (200 µg/ml; InvivoGen, ant-hg-5) and a FACS sorting procedure (FACSAria Fusion, BD Biosciences) based on GFP fluorescence.

### Transient transfection

Cells were transfected the day before the experiment using a Neon transfection device (Thermo Fisher Scientific) according to the manufacturer's instructions. MCF-7 parameters were: pulse voltage 1250v; pulse width 20 ms; pulse number 2. C-26 parameters were: pulse voltage 1350v; pulse width 20 ms; pulse number 2. pcDNA3.1-mCherry was from Addgene (Addgene plasmid #128744, RRID:Addgene_128744; deposited by David Bartel).

### Imaging settings

Image acquisition was performed with a Zeiss LSM880 inverted confocal microscope objective and using Zeiss ZEN2.3 SP1 FP3 (black, version 14.0.24.201), using a 63×1.46 NA oil immersion objective and a 403, 488, 561 and 633 nm wavelength lasers, in the Fast Airyscan mode. Voxel size was of 0.1413 µm for *xy* and *z*-step of 0.4 µm. This allowed us to activate the Definite Focus system, so the sample was autofocused every time frame. Pixel sizes are of 0.1409 µm, and *z*-spacing for the objective is 0.4 μm, which turns into 0.3440 µm after correction. *Z*-spacing was corrected following the literature (Diel et al., 2020), considering the cell refractive index of 1.36 and Immersol immersion oil of 1.518.

For cell layers, image positioning was automatically set to fit a tile positioning with an 15% image overlap. In the case of YAP immunostaining for cell layers, only properly permeabilized regions were imaged. To recognize the properly permeabilized regions a control staining of Sencyt was performed (not shown).

### Osmotic shock experiments

#### Cell seeding

Single-well, Mattek, glass-bottom dishes were incubated with 10 μg/ml of fibronectin (Sigma-Aldrich) in PBS for 1 h at room temperature. Cells were seeded on the plate to achieve an approximate density of 1000 cells/mm^2^ the day after. At a minimum of 1 h prior to experiment, the medium of the cells was changed to 500 µl of medium containing 1:10,000 Hoechst 33342 (Invitrogen).

#### Image acquisition and osmotic shock

The time frame was set to 30 s; for every sample, we imaged five timepoints without disturbances before the shock was applied. Then we imaged for 45 timepoints more. To decrease image drift while acquiring images of the same position through time, we started the imaging of the sample with 0.5 ml of medium containing the nuclei stain. At the time of the shock, we added 1 ml of 1.5× solution either for hypo- or hyper-osmotic shock conditions. The control worked as an imaging control condition.

Cell medium has an osmolarity of ∼340 mOsm. ∼113 mOsm hypo-osmotic shocks (66%) were performed by mixing the 500 µl of medium with 1.5× de-ionized water with Ca^2+^ and Mg^2+^ ion concentration corrected to match those of the medium (264 mg/l CaCl_2_·2H_2_O, 164.67 mg/l MgCl_2_·6H_2_O). ∼695 mOsm hyper-osmotic shocks (204%) were performed by adding 1 ml of 1.5× solution containing 96.9 g/l D-mannitol (Sigma) to the medium.

For the analyses, *t*=30 s was discarded because it was noisy due to out of focus imaging after the medium pipetting.

### Drug treatment experiments

#### Cell seeding

Six-well glass-bottom dishes (Mattek) were incubated with 10 μg/ml of fibronectin in PBS for 2 h at room temperature. Cells were seeded on the plate in mediums containing 1:10,000 Hoechst 33342 (Invitrogen) and either the drug vehicle (3.5 µl DMSO/1 ml of medium) or a combination of the drugs [25 µM para-NitroBlebbistatin (Sigma-Aldrich), 50 µM CK666 (Sigma-Aldrich)]. Cells were left to attach to the substrate for 2 h before the imaging started.

#### Image acquisition and drug washout

Image acquisition parameters were performed in an identical manner to the osmotic shock experiments unless specified otherwise. For the drug washout experiment, cells were imaged every hour, starting 2 h after seeding. For two timepoints cells were left untouched. After the imaging of the second timepoint finished, we aspirated the drug-containing medium, washed twice with warm medium, and added the vehicle-containing medium for the following timepoints.

### Cell layer experiments

#### Cell seeding

Glass-bottom dishes (Mattek) were incubated with 10 μg/ml of fibronectin in PBS for 2 h at room temperature. Magnetic PDMS gaskets (Rodriguez-Franco et al., 2017) sized 4 mm times 8 mm at the inner side, were treated water and soap, washed in ethanol, washed in MiliQ water, incubated in Pluronic^®^ F-127 (20 g/l) for 1 h at room temperature, washed twice in PBS, and air dried. Both Mattek dishes and gaskets were UV sterilized before seeding. For cell seeding, gaskets were put in the center of the Mattek dishes, and the dishes were placed on top of a holder including a magnet to keep them in place. ∼60,000 cells were seeded in every gasket (0.3 cm^2^). Cells were incubated for 4 h, and then some washes with medium were performed to retrieve non-attached cells. Enough medium was added to cover the gaskets completely. Cells were then incubated for 24 h with the gasket. The gasket was then retrieved, and cells were incubated overnight before imaging started.

#### Staining

Immunostainings were performed as previously described (Elosegui-Artola et al., 2017). Cells were fixed with 4% (v/v) paraformaldehyde for 10 min, permeabilized and blocked with 0.1% (MCF-7) and 1% (C-26) (v/v) Triton X-100 and with 2% (v/v) fish-gelatin (Sigma-Aldrich) in PBS once for 45 min, incubated with primary antibody for 1 h at room temperature or overnight at 4°C, washed three times with fish-gelatin in PBS for 5 min, incubated with secondary antibody for 1 h, washed with fish-gelatin in PBS three times for 5 min, and imaged in PBS with the same conditions as for the live imaging. YAP mouse monoclonal antibody (cat. no sc101199; RRID: AB_1131430), and secondary Alexa Fluor-555 (Thermo Fisher Scientific, goat anti-mouse-IgG, A-21424; RRID: AB_141780) were used diluted 1:400.

#### Image analysis

Images were processed to .czi format with Zeiss ZEN2.3 SP1 FP3 (black, version 14.0.24.201). Then they were binned in *xy* by a factor of 4 calculating the median using Fiji software (Schindelin et al., 2012), leaving the voxel size in *xy* at 0.5652 µm (*z* remained untouched, values were averaged) then they were separated by channels (the nuclei staining was filtered with a median filter of 2 pixels for osmotic shock experiments, to decrease the effects of chromatin staining changes into segmentation). The processed nuclei image was then segmented in 3D using Cellpose (Stringer et al., 2021), which is described by the developers as a generalist algorithm for cellular segmentation. It was developed by training the algorithm of thousands of images via deep learning, making it an easy-to-use and robust piece of software for segmenting cellular structures.

Parameters for Cellpose were set as follows:

Osmotic shocks experiments: *python -m cellpose --dir* [directory] *--do_3D --cellprob_threshold=-2.0 --batch_size 2 --pretrained_model nuclei --chan 1 --diameter 34. --save_tif --no_npy --use_gpu --verbose --anisotropy 0.6*.

Drug washout experiments: *python -m cellpose --dir* [directory] *--do_3D --cellprob_threshold=-2.0 --batch_size 2 --pretrained_model nuclei --chan 1 --diameter 34. --save_tif --no_npy --use_gpu --verbose*

Cell layer experiments: *python -m cellpose --dir* [directory] *--do_3D --cellprob_threshold=0.0 --batch_size 2 --pretrained_model nuclei --chan 1 -- diameter 34. --save_tif --no_npy --use_gpu --verbose*

Using the masks created by Cellpose, we measured fluorescent intensities inside and outside of the nucleus for the plane of biggest area for every nucleus. The nuclear area was created by eroding this plane by 1 pixel (0.5652 µm), and the cytoplasmic area by creating a ring outside the nucleus. This was undertaken by subtracting a 3-pixel-increased area, by a 1-pixel-increased area. Then any pixel in the cytoplasmic area was excluded if it fell inside any neighboring nucleus. This was done for all channels, as well as measuring geometrical and size parameters of the masks using a script written in MATLAB (2020b) (available upon request). To avoid spurious measurements some filters were applied. For cell brightness: a minimal signal to noise ratio filter was applied. To prevent dim cells next to a very bright cell, or vice versa being analyzed, all measurements of areas with a coefficient of variation higher than 0.8 were discarded. To prevent cells with bad nuclei segmentation being analyzed, nuclei with nuclear-to-cytoplasmic brightness ratios for Hoechst 33342 lower than 4 were discarded.

#### Data quantification and parameters

Once the nuclei masks were obtained, we calculated the different nuclear shape parameters using a script written in MATLAB (available upon request) (see Fig. 2) in two ways: directly from the mask (volume abd Solidity index) and fitting an ellipsoid and obtaining the length of the three radii (oblateness and prolateness). In the case of the Solidity index, the convex hull volume is the smallest convex volume that contains a shape.

The Sencyt index was calculated as the logarithm in base 2 of the ratio of the mean nuclear fluorescence (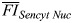) and the mean cytoplasmic fluorescence (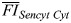) of Sencyt, after subtracting mean background fluorescence (
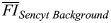
, assessed in cell-free regions of the image):

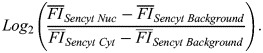
The log2 nuclear-to-cytoplasmic YAP ratio was calculated as the logarithm in base 2 of the ratio of the mean nuclear fluorescence (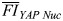) and the mean cytoplasmic fluorescence (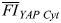) of YAP staining, also after subtracting mean background fluorescence (
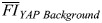
):

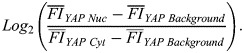
For the Solidity index, sphericity, oblateness and prolateness, see Fig. 2. Nuclear and cytoplasmic regions were determined as explained in the ‘image analysis’ section above. For the cytoplasm, regions immediately adjacent to the nucleus were used.

Cell density was calculated with the *xy* coordinates by measuring the number of nuclei around the *n*th nucleus. This was done by centering a square in the *n*th nucleus with a side size of 200 pixels, which is 113.03 µm.

For Fig. 6E,K, direct nuclei correlation between the live Sencyt images and the YAP staining images was undertaken by nuclei image registration and overlap of the masks. This way we obtained a table with the corresponding mask identifiers in live and staining images.

### E-cadherin staining

#### Cell seeding

Glass coverslips (#1.5, 25 mm diameter, Menzel-Gläser) were coated with 10 μg/ml fibronectin (bovine plasma, MERCK/Sigma, F1141) overnight. Coverslips were washed with PBS (Gibco 14200-067) twice and with cell medium before cell plating. ∼5000 cells/mm^2^ were plated in the center of each fibronectin-coated area to obtain samples with different densities within the same area. Samples were washed after 1 h to remove not-attached cells and cultured for 24 h before fixation (4% paraformaldehyde solution in PBS, Santa Cruz Biotechnology, sc-281692).

#### Staining

Immunostaining was performed as described for cell layer experiments except for the following. Primary human E-cadherin mouse antibody (Cat. no. 610181, BD Pharmingen, clone 36/E-Cadherin) was diluted 1:500 and secondary Alexa-647 donkey-anti mouse-IgG (Thermo Fisher Scientific, ref. A31571) at 1:1000 and incubated overnight. After the secondary antibody was removed and cells were washed three times with PBS and stained with phalloidin–TRITC (555, ref. P1951-0.1MG, Sigma-Aldrich) at 0.1 μg/ml diluted in PBS for 1 h and with Hoechst (Hoechst 33342, trihydrochloride, trihydrate - 10 mg⁄ml solution in water; Invitrogen H3570) at 0.5 μg/ml for 10 min. Samples were then washed three times in PBS, dried and mounted in MOWIOL (4-88 Reagent Calbiochem-Merck, 475904) by letting the coverslip dry overnight on top of a standard microscopy slide.

#### Image acquisition

This was as described in the Imaging Settings unless specified differently. Pixel and voxel sizes were set to the optimal values of the Airyscan. Accordingly, voxel size in *xy* was equal to 0.071 μm and to 0.159 μm in *z*. As the sample was in MOWIOL, which matches the oil refractive index, no *z* correction was applied to these data.

### Micropatterning experiments

#### PRIMO

Fibronectin circular micropatterns were obtained by using light-induced molecular adsorption of proteins (LIMAP) (Strale et al., 2016) with the PRIMO device (Alvéole). PRIMO was coupled to an inverted microscope Nikon Eclipse Ti-e equipped with a 20× NA 0.45 objective.

In brief, we first prepared polyethylene glycol (PEG)-coated glass-bottom dishes (Mattek). To this end, the glass surfaces from the dishes were plasma treated using a Corona plasma cleaner device and subsequently covered with a 0.5 mg/mL polyline(PLL)-g-PEG {PLL (20 kDa)-g[3,5]-PEG (2 kDa), Alveole Ref PLLgPEG; 10 mg} in PBS for 1 h. The plates were subsequently rinsed three times with PBS. Before micropatterning, the PBS was removed and replaced by a 14 mg/ml solution of the photoinitiator PLPP (Alvéole, BOC Sciences CAS no. 78697-25-3), which triggers the photo-scission of PEG upon UV illumination. A pattern of UV light (1200 mJ/mm^2^) containing circles of diameters of 10, 20, 30 and 40μm was projected through the glass surface. After UV illumination, the plates were washed three times with PBS, incubated with a 10 μg/ml fibronectin solution in PBS for 10 min and finally washed again three times with PBS. Plates were stored filled with PBS at 4°C until further use (a maximum of 1 day after patterning).

#### Cell seeding

Plates were rinsed in cell medium twice and ∼30,000 cells (single-cell suspension) were added to each plate. C26 or MCF7 cells were incubated for 1 h to make them adhere to the patterns, then nonattached cells were washed out and cells were incubated for 1 h to reach full cell spreading. Cells were then stained with 0.5 μg/ml Hoechst 33342 for 10 min.

#### Image acquisition

This was as described in the in Imaging Settings unless specified differently. Pixel and voxel sizes were set to the optimal values of the Airyscan. Accordingly, voxel size in *xy* was equal to 0.071 μm and to 0.159μm in *z*, which is then rescaled according to the refractive index mismatch to 0.137μm.

#### Image analysis

For the nucleus shape analysis, cells were segmented using Fiji software and the masks uploaded into MATLAB to perform the same 3D nucleus shape analysis. Nuclei with masks exceeding top or bottom slices of the acquired stack were excluded for 3D shape analysis. For the Sencyt and YAP ratios, the cross section of nuclei was segmented in Fiji software using the DNA (Hoechst 33342) channel. This allowed the definition of the nuclear area (Hoechst mask, reduced by 3 pixels) and cytosolic area (defining a 3 pixel ring outside the Hoechst mask). The mean fluorescence intensity of YAP or Sencyt and background intensities were measured directly in Fiji software. As cells attached to small micropatterns sometimes moved during the image acquisition, poor overlap in between the DNA (Hoechst 33342) and Sencyt channels could be observed. In these cases, the regions had to be manually corrected. All segmentations were post-checked manually.

## Supplementary Material



10.1242/joces.262363_sup1 Supplementary information
