# Supplementary material for: Nucleocytoplasmic transport senses mechanical forces independently of cell density in cell monolayers
Source: J Cell Sci. 2024 Sep 9;137(17):jcs262363. doi: 10.1242/jcs.262363 (PMC11423809; doi:10.1242/jcs.262363)
Supplement: Supplementary information [file joces-137-262363-s1.pdf]

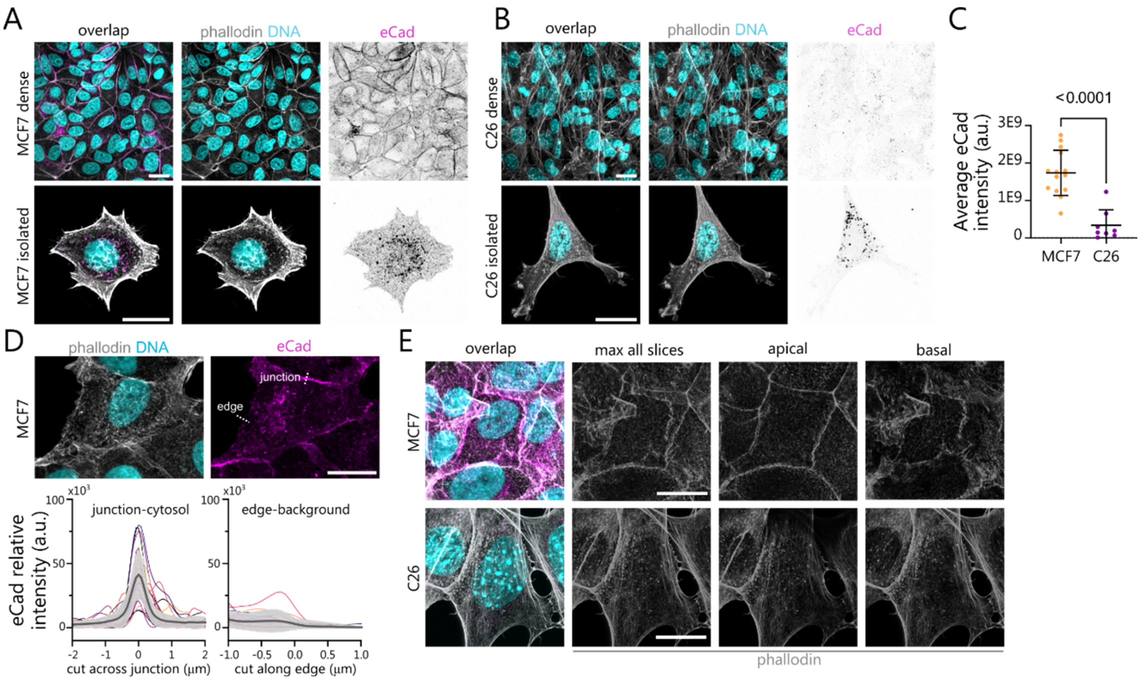

Fig. S1. E-Cadherin accumulates at cell-cell junctions in MCF7 cells only. A) Representative maximum projection images of fixed connected/dense MCF7 cells (top) and of an isolated MCF7 cell (bottom) stained with phalloidin, DNA-Hoechst and eCadherin. B) Representative maximum projection images of fixed connected/dense C26 cells (top) and of an isolated C26 cell (bottom) stained with phalloidin, DNA-Hoechst and eCadherin. C) Quantification of total eCad intensity per field of view normalized per number of cell nuclei for C26 and MCF7. Black lines represent mean +- SD. Statistical difference in between the two groups was assed using a non-parametric Mann-Whitney test. (MCF7 N= 14 fields of view; C26 N= 8 fields of view) D) Top: Z-projection (sum slices) of MCF7 cell stained with phalloidin, DNA-hoechst and eCadherin with representative white lines indicating a cell-cell junction and a cell-edge boundary. Bottom: Relative fluorescence eCadherin intensity profile at cell-cell junctions (relative to the cytosolic value, left) and at the edge of a cell (relative to the cell-free background, right). Junction intensity profiles are aligned at the maximum while edge profiles aligned with respect to the step in intensity. Each line corresponds to an intensity profile from one cell (N=51, 36). Thick grey lines represent the mean and the shaded areas the standard deviation. E) Representative images of MCF7 cells (top) vs C26 cells (bottom) stained with phalloidin, DNA-Hoechst and eCadherin. From left to right: maximum projection of the 3-channels overlap, maximum projection of the phallodin channel, maximum projection of the most-apical phalloidin slices, maximum projection of the most basal phalloidin slices. MCF7 cells shows apical ruffles and basal stress fibers while C26 cells show apical and basal stress fibers. All scale bars 20µm.

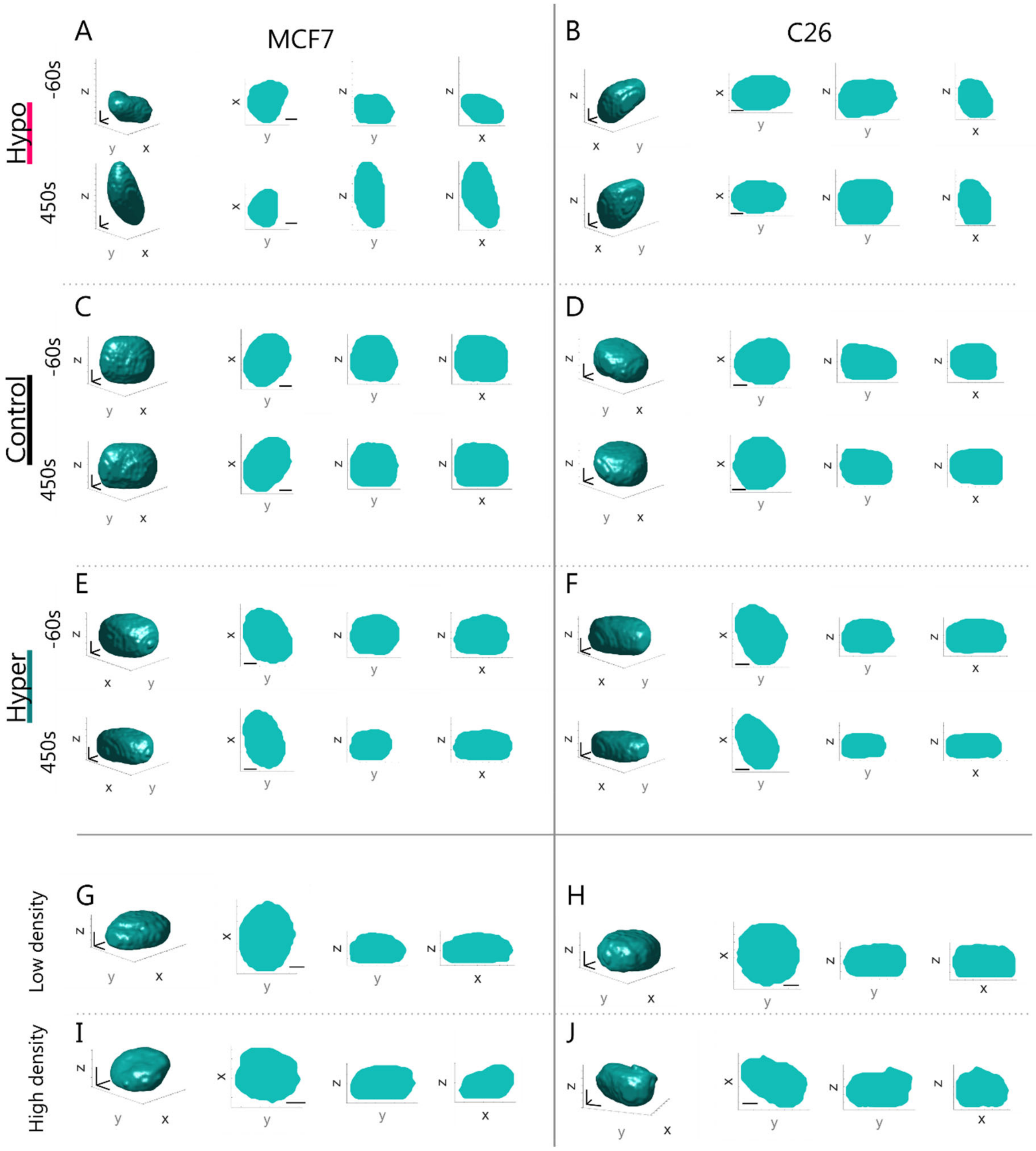

Fig. S2. A-F) Extended views of 3D rendering of example nuclei in Fig. 1. G-J) Extended views of 3D rendering of example nuclei in fig. 5. 3D view plus xy zy zx cuts. Scale bar 4μm.

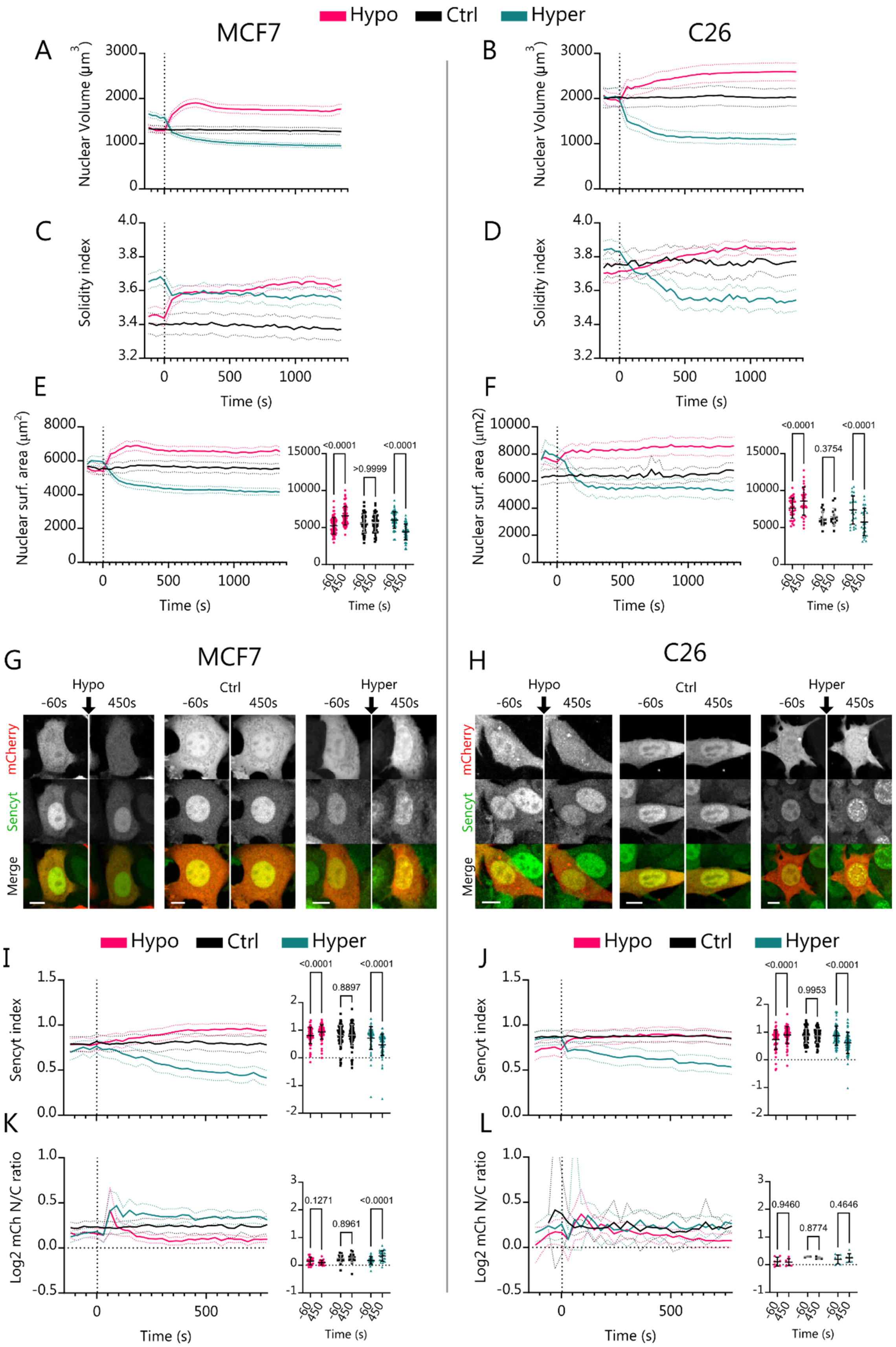

Journal of Cell Science • Supplementary information

Fig. S3. Additional measurements of effects of osmotic shocks. Absolute values for Nuclear Volume (A-B) (N=170, 130, 188, 81, 72, 107 cells) and Solidity index (C-D) (N=230, 170, 231, 121, 104, 142 cells), for MCF7 and C26, respectively. E-F) Change of nuclear surface area over me for MCF7 and C26, with corresponding sta s cs (N=68, 50, 59, 32, 15, 26 cells). G-H) Representative images of cells transfected with mCherry, submitted to osmotic shocks as in Fig. 1. Scale bar is 10 µm. I-J) Corresponding quantification of Sencyt index (N=71, 78, 73, 58, 67, 69 cells) and K-L) Log2 mCherry Nucleo-cytoplasmic ratio (N=20, 20, 27, 7, 3, 7 cells). p-values calculated with 2-way ANOVA corrected with Šídák's multiple comparisons test. Error bars represent 95% CI for melapse graphs and SD for statistical graphs. All data include 3 independent repeats.

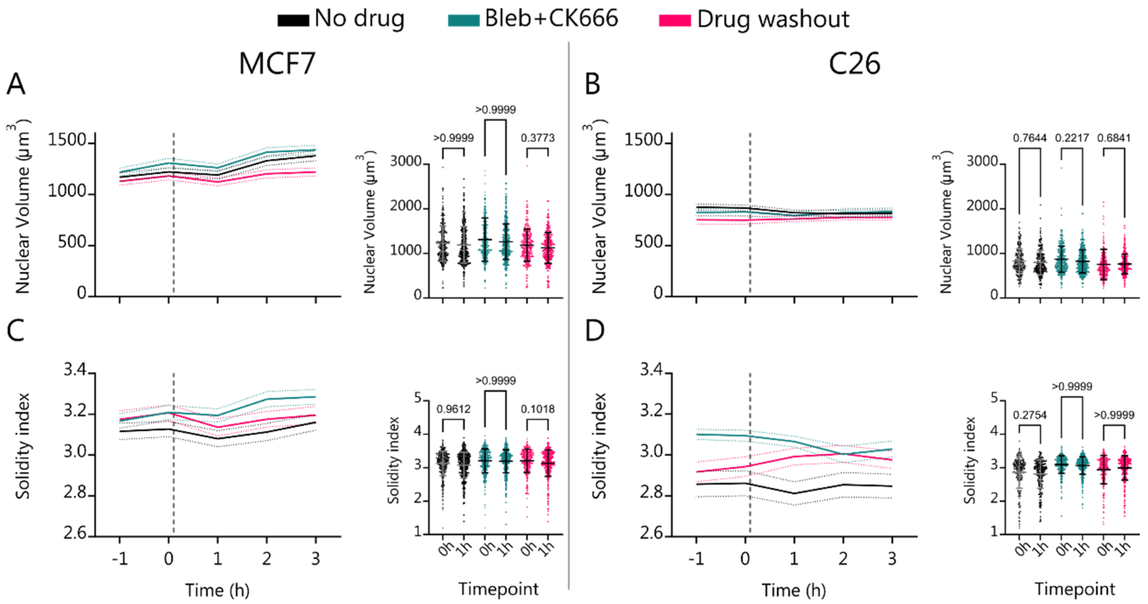

Fig. S4. Nuclear volume and Solidity index values corresponding to Fig. 3 data. A-B) Nuclear Volume (N=368, 370, 398, 436, 335, 332, 230, 218, 368, 394, 303, 302 cells) and C-D) Solidity index measurements and statistics for MCF7 and C26 as a function of time. (N=368, 370, 398, 436, 335, 332, 230, 218, 368, 394, 303, 302 cells). p-values calculated with Kruskal-Wallis test corrected with Dunn's multiple comparisons test. Error bars represent 95% CI for timelapse graphs and SD for statistical graphs. All data include 3 independent repeats.

Journal of Cell Science • Supplementary information

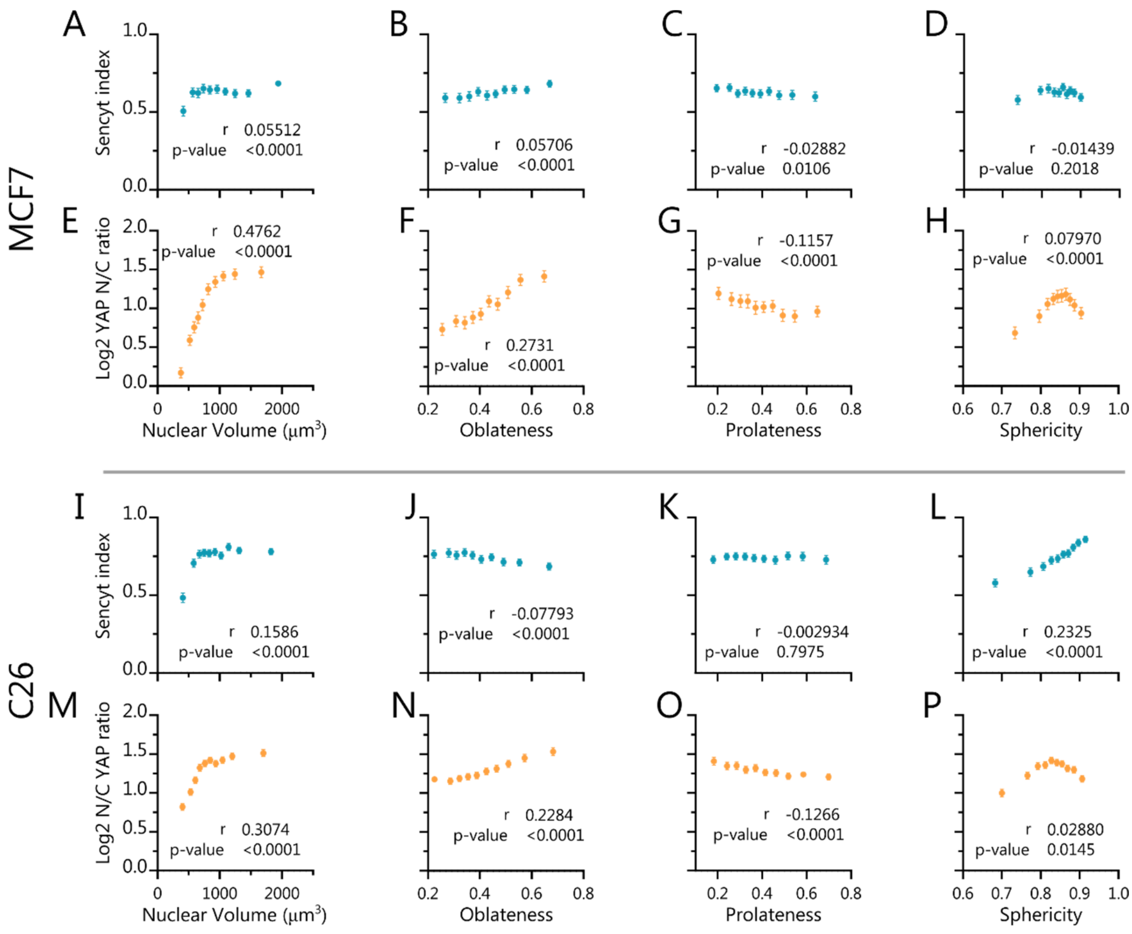

Fig. S5. Sencyt index and YAP localization versus nuclear shape parameters. (A-D N=7865 cells, E-H N=4889 cells, I-L N=7647 cells, M-P N=7204 cells). p-values calculated with Two-tailed non-parametric Spearman correlation test. Error error bars represent 95% CI. All data include 3 independent repeats.

Journal of Cell Science • Supplementary information

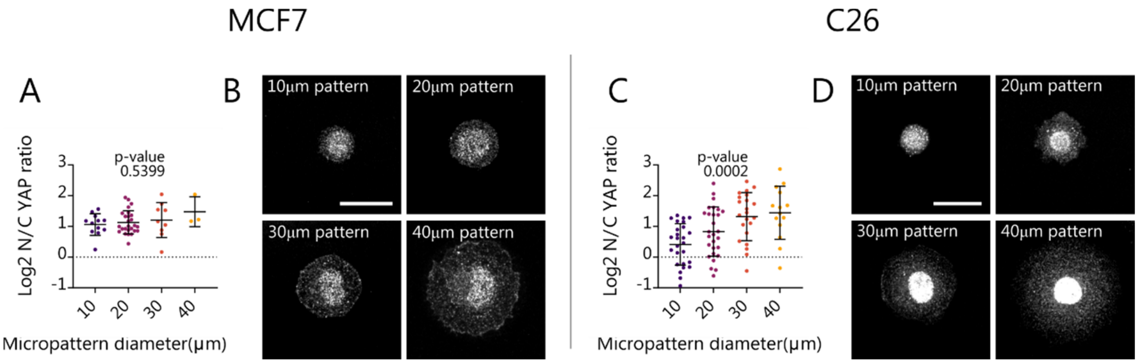

Fig. S6. A,C) YAP nucleus to cytosolic ratio for MCF7 and C26 cells cultured on cultured in circular patterns of indicated diameters. (MCF7 N= 12, 26, 9, 3 cells; C26 N= 26, 28, 22, 14 cells) B,D) Representative maximum projection images of fixed C26/MCF7 cells cultured in circular patterns of indicated diameters and stained for YAP. Scale bars 20 µm. Black lines represents mean and standard deviations. p-values are calculated using non-parametric Kruskal Wallis test. All data include 3 independent repeats.

Journal of Cell Science • Supplementary information
