# Supplementary material for: Nucleocytoplasmic transport senses mechanical forces independently of cell density in cell monolayers
Source: J Cell Sci. 2024 Sep 9;137(17):jcs262363. doi: 10.1242/jcs.262363 (PMC11423809; doi:10.1242/jcs.262363)
Supplement: Supplementary file 1 [file joces-137-262363_review_history.pdf]

Nucleocytoplasmic transport senses mechanical forces independently of cell density in cell monolayers

Ignasi Granero-Moya, Valeria Venturini, Guillaume Belthier, Bart Groenen, Marc Molina-Jordán, Miguel González-Martın, Xavier Trepat, Jacco van Rheenen, Ion Andreu and Pere Roca-Cusachs
DOI: 10.1242/jcs.262363

Editor: Andrew Ewald

Review timeline

Submission to Review Commons:	10 January 2024
Submission to Journal of Cell Science:	18 June 2024
Editorial decision:	15 July 2024
First revision received:	23 July 2024
Accepted:	24 July 2024

Reviewer 1

Evidence, reproducibility and clarity

Summary

The nucleus is recognised as a core component of mechanotransduction with many mechano-sensitive proteins shuttling between the nucleus and cytoplasm in response to mechanical stimuli. In this work, Granero-Moya et al characterise a live florescent marker of nucleocytoplasmic transport (NCT) and how it responds to a variety of cues. This work follows on from the authors previous study (Andreu 2022) where they examined the response of passive and active NCT to mechanical signalling using a series of artificial constructs. One of these constructs (here named Sencyt) showed a differential localisation depending on substrate stiffness, accumulating in the nucleus on stiffer substrates (which the authors previously showed was due to differences in mechano-sensitivity of passive versus facilitated NCT). Here the authors use Sencyt as a tool to probe how different cues affect NCT and thus nuclear force-sensing in two different cell lines (one epithelial, one mesenchymal).

They have established a 3D image segmentation pipeline to measure both the nuclear/cytoplasmic ration of Sencyt and 3D nuclear shape parameters. As a proof-of-principle, they show that hypoosmotic shock (which inflates the nucleus and would be expected to increase nuclear tension) and hyper-osmotic shock (which shrinks and deforms the nucleus) alter Sencyt nuclear-cytoplasmic ration as expected. They then show that inhibiting acto-myosin, which would be expected to block force transduction to the nucleus, reduces NCT, although interestingly this is without any changes to nuclear morphology. They then examine how cell density affects NCT and show that Sencyt localisation correlates only weakly with density but much more strongly with nuclear deformation (especially as measured by solidity). This is surprising considering that mechano-sensitive transcription factors such as YAP have been shown to exit the nucleus at high cell densities. Therefore, the authors directly compare Sencyt and Yap nucleo/cytoplasmic localisation and show that Sencyt behaves differently to YAP with YAP localisation correlating strongly with cell density. This reveals an added layer of complexity in YAP regulation beyond pure changes to NCT.

Major points

© 2024. Published by The Company of Biologists under the terms of the Creative Commons Attribution License (https://creativecommons.org/licenses/by/4.0/).

The data presented throughout this work are high quality and rigorous. The controls used are appropriate (including the use of a freely diffusing mCherry to illustrate the specificity of the Sencyt probe in osmotic shock experiments - figure S2). Experiments are properly replicated and the statistical analysis is appropriate. The data are beautifully presented in figures and the manuscript is well written and very clear. Overall this is a high quality work.

The discussion is careful and the conclusions are supported by the data. My only small concern is that the authors place too much emphasis on how this work is in 'multicellular systems' as opposed to their previous work in single cells (for example "Here, we demonstrate that mechanics also plays a role in multicellular systems, in response to both hypo and hyper-osmotic shocks, and to cell contractility. L212). Cell density is only controlled in figures 3 and 4 and in some of the earlier experiments, cells look quite sparse (eg Figure 2). It's also debatable how far a monolayer of cancer cells, which lack contact inhibition of growth, is a multicellular system. Furthermore, the authors don't specifically look at cell/cell adhesion or observe major differences between the epithelial or mesenchymal lines. For this reason, the authors should tone down this discussion before publication.

Optional experimental suggestions: For me, the most compelling finding is that nuclear deformation has a greater correlation with NCT than cell density and that this is different from the behaviour of YAP. To cement the importance of nuclear deformation, the authors could induce deformation in single cells, for example by culture on very thin micropatterned lines and assess the localisation of Sencyt and YAP. It would also be interesting to assess the role of force transduction in this context or in different densities by removing actin, which affects NCT without inducing nuclear shape changes. These functional experiments would allow the authors to draw stronger conclusions about the role of nuclear shape and deformation but they aren't necessary for publication.

Minor points

- I'd like to see better examples of 3D reconstructions of nuclei (ie fig 1C but bigger) in different conditions. This is especially important in figure 3 where it would be helpful to see examples of nuclei with high or low solidity. The differences in oblateness are clear to see from the images in 3a and 3f but solidity could be better illustrated.
- Where Sencyt index is plotted, it would be clearer to add labels to at least figure 1 indicting which indicate whether it is more cytoplasmic or nuclear.

Referees cross-commenting

Here comments from all 3 reviewers are reported

Reviewer 1:

I disagree with R2's comment that there is 'no novelty' here. Although this work is going to be of greater interest to a specialised rather than general audience, it characterises in depth a simple tool to measure NCT which will be useful for mechanobiology field. Also, using 'two cellular model systems in vitro' is very standard in the field when assessing subcellular processes like NCT. Using this approach in vivo would be very interesting but challenging and would be an entirely different study.

I agree with R2's comments that the authors should better justify their combination of two actin inhibitors and R3s point on better assessing cell/cell junctions.

Reviewer 2

About Reviewer 3's comments, I believe it's a stretch to highlight the strength and novelty based on "NCT's mechanosensitivity in epithelial cells has not been demonstrated,". There are thousands of papers on the Hippo pathway, that is known to be mechanosensitive, on the regulation of YAP, that enters in the nucleus in Hippo inhibited conditions and exits to the cytoplasm in Hippo induced cells, including downstream of mechanical signals. The phenomenon of nuclear-cytoplasmic shuttling being a common event from neurons to

© 2024. Published by The Company of Biologists under the terms of the Creative Commons Attribution License (https://creativecommons.org/licenses/by/4.0/).

endothelial and multiple types of epithelial, immune, and fibroblast cells is already established through NCT of this and other endogenous proteins. This is simply an accepted fact.
Then, The Nature cell Biology 2022 was offering a very general claim. No warning that conclusions could have been cell type specific. In the Artola 2017 Cell paper they also showed NCT in mammary epithelial cells. We should definitively conclude that NCT's mechanosensitivity in epithelial cells has been well demonstrated.

About Reviewer 1: I find it challenging to grasp the point made in the comment. On novelty, in their previous study in NBC 2022 Syncet was already shown to undergo NCT.
The reviewer states that the study presents "a simple tool to measure nuclear-cytoplasmic transport (NCT) beneficial for the mechanobiology field, and evidence that this demonstrates a novel layer of regulation in hippo signaling (also because this is observational and not a mechanistic study). The tool in question is far from simple. Its application requires transfection into cell cultures, conducting live imaging, etc. If one aims to measure NCT of endogenous proteins, straightforward immunofluorescence or live imaging of endogenous proteins (like GFP-tagged YAP, Twist, Smads, etc.) using the same experimental setup should suffice to demonstrate relevance, without necessitating any additional experiments. What then, is the unique benefit of this proposed tool? Given it's an artificial construct combining NLS-GFP with a bacterial protein, questions arise about the effects of the forced nuclear localization signal (NLS) or the bacterial component. It is an empirical artificial construct and there is no mechanism to explain its behavior.
The comparison of Syncet with YAP seems to me questionable and of limited utility. It's unsurprising that an artificial construct only mirrors some aspects of what is considered a genuine mechanosensitive protein. The utility of a synthetic tool lies in its ability to replicate actual phenomena, not in what it fails to do. In comparison to their NBC 2022 study, this manuscript focuses on what their reporter fails to detect. The osmotic shock was the assay in their 2017 Cell paper.
Here they demonstrate that a combination of Blebbistatin+CK (an unclear choice of drugs) is ineffective, as is cell density. Are there other specific peculiarities associated with this construct?

My other concern is on the minor quantitative changes reported, which seem inconsistent with the provided representative images, where significant differences are difficult to appreciate. For instance, the claim that the transfected sensor differs from an endogenous NCT protein, YAP, after cell density treatment, is hard to detect in their images. In Figure 4, comparing YAP and Syncet in C26 cells, YAP appears uniformly nuclear at high cell density, potentially more nuclear than the synthetic sensor, which is not coherent with their claim.
From the Hippo perspective, there is really an unusual amount of nuclear YAP left in their cells. This should be almost completely cytoplasmic from prior contact inhibition studies in the Hippo field. Syncet could be simply less sensitive than YAP in these borderline conditions. Although there's a more noticeable cytoplasmic noise in dense cells with YAP compared to Syncet, this could be attributed to several factors, including differences in protein degradation rates, which I suspect to be quicker for a synthetic protein. From a technical perspective it is complex to get strong conclusions after comparing something so unrelated with each other. One is a live GFP detection and the other is a staining by immunofluorescence. the nature of the background is also different and so conclusions from comparisons between unrelated systems is not justified.
This suggests caution on what is heralded as the main claim here put forward.

Reviewer 1:

I do have some sympathy with R2s comments in the consultation. I agree that showing that NCT is mechanosensitive in an epithelium is not new. I also agree that sometimes it is difficult to see the quantitative differences by eye. This second point could be addressed by including more details of the segmentation and analysis in the supplemental material (along with some example images).

Regarding novelty, I would be interested to know if R2 thinks that there are experiments that the authors could do to improve the work. Or do they need to simply tone down their claims?

© 2024. Published by The Company of Biologists under the terms of the Creative Commons Attribution License (https://creativecommons.org/licenses/by/4.0/).

It's perfectly acceptable to publish a well characterised tool with a series of observations and it's beneficial to the community to do so.

Reviewer 3

Thanks to Reviewers #1 and #2 for using this consultation option; I truly appreciate their feedback on my comments and find it extremely valuable.
I agree with Reviewer #1 that the method proposed here is relatively simple. Transfecting cells and conducting live fluorescent imaging can hardly be considered difficult. I believe the construct used/designed by the authors is the main advantage as it provides a specific way to quantitatively assess NCT and not limit the analysis to a single nuclear protein (such as YAP). Reviewer #2 suggests using immunofluorescence staining of YAP or live imaging of fusion fluorescent protein (following transfection) to analyze NCT, but this approach would yield a readout not only based on NCT but also on the many other interacting partners/mechanisms that regulate the candidate localization, resulting in an unspecific readout (and similar transfection/live imaging set-up).
Regarding the impact of the study, I agree that it is certainly not as impactful as previous publications on this topic. Although I find reviewer#2 argument on Yap irrelevant, as YAP is not the main focus of this paper. Some experiments have been done with cells of epithelial origin, but NCT mechanosensitivity has not been clearly tested in epithelial monolayer, which is the main claim of the proposed study here. The 2017 Cell paper focused on YAP transport into the nucleus (and not NCT in general) and they showed a correlation between YAP nuclear localization and traction force in MCF10A. I am not sure if one would say that "NCT mechanosensitivity has been well demonstrated in epithelial cells" based on this single panel. The impact of the proposed study is certainly not outstanding but offering a thorough analysis in epithelial cells (as monolayers and not as individual cells) and presenting a well- defined experimental approach should be of interest in the field.
I agree with comments from reviewer#2 that some reported effects in graph are unclear on main images. More experimental details should hopefully clarify this aspect.

Significance

In this work, Granero-Moya et al characterise a new tool for measuring NCT and show that it is mechanically regulated. Given the importance of NCT in mechano-transduction, this tool will be a great asset to the mechano-biology community and will likely be adopted by multiple groups in the future. The findings about the effects of cell density on NCT and differences from YAP are interesting but could be further fleshed out. This work is likely to be of greatest interest to a specialised audience working in the fields of mechano-biology and nuclear transport.

Reviewer 2

Evidence, reproducibility and clarity

The study conducted by Granero Moya and colleagues describes the application of a synthetic protein which is observed to enter the nucleus in response to mechanical strains, rather than being influenced by cell density. However, the novelty of this work is minimal since the conceptual framework and the utilization of this identical or similar tool have been previously reported by the same team in earlier publications. In their experiments, they employ this GFP-based sensor, referred to as Sencyt, in cells subjected to osmotic shocks. These shocks are highly stressful and impact a range of cellular processes, including stress response pathways MAPK and others; Osmoregulatory pathways; cell cycle regulations, autophagy and death pathway; ion channel regulations and others.
The second findings are on cells treated with a combo of drugs affecting the actin cytoskeleton. The justification for using a combination of two specific drugs remains unclear, as the study does not adequately explain the rationale behind this choice. Additionally, there is a lack of information regarding the full range of targets these drugs affect. This raises questions about the comprehensiveness and applicability of the findings, as understanding the

© 2024. Published by The Company of Biologists under the terms of the Creative Commons Attribution License (https://creativecommons.org/licenses/by/4.0/).

complete scope of the drugs' targets is crucial for interpreting the results within a minimal frame of physiological context.

The novelty is on the specificity of this synthetic fusion protein for these manipulations and not on cell density. Yet, the reasons behind this selective response remain unexplained, potentially attributable to the unique characteristics or sensitivity thresholds of their synthetic probe. As comparison, YAP localization and this is sensitive to both inputs, but this is also already published (fig4). The focus is anyway on Sencyt for which they offer simple observations and quantifications. When reviewing the figures presented, I find it challenging to detected marked differences, despite their quantitative data suggesting otherwise. Furthermore, the study attempts to correlate the behavior of Sencyt with the nuclear geometric parameter of solidity, a connection that seems to lack a clear basis in cell biology and could potentially lead to misconceptions.

Significance

In sum, I think the MS is of interest for a very specialistic audience. There are no clear interpretations. The work is done in one or two cellular model systems in vitro; and the general significance of these observations is of very limited impact and no novelty.

Reviewer 3

Evidence, reproducibility and clarity

In this very well-written manuscript, Pere Rochas-Cusachs and colleagues investigated the response of nucleocytoplasmic transport (NCT) to mechanical stress and tested whether this response is similar in epithelial and mesenchymal cells using a combination of quantitative approaches. This study builds upon their earlier findings, which elegantly demonstrated that NCT is sensitive to mechanical forces transmitted to the nuclear membrane. Using a similar approach to their recent work, they quantitatively analyzed NCT and compared the two cell types using various treatments that impact nuclear membrane tension. The study is straightforward and experimentally sound, with an adequate number of replicates and independent experiments. While one might consider the limitations given their previous work, none have demonstrated that NCT is mechanosensitive in epithelial cells. Additionally, they provide a simple approach to measure NCT, which should be of interest in the field. However, it is unclear how the authors defined the epithelial phenotype in this work and whether they solely based this characterization on the tissue/cell's origin. Epithelia can be defined ultrastructurally with reference to their apico-basal polarity and specific cell-cell junctions (Alberts et al., 1994; Davies and Garrods, 1997). Changing cell density should affect cell/cell adhesion, but the authors provide no evidence that the cells tested in the study are attached to their neighbors on all sides and form an epithelium. While I recognize that the objective of this study is not to mimic the in vivo behavior of epithelial tissue, the authors should at least ensure that cells form a monolayer by quantitatively assessing cell- cell junctions (or they should adjust their conclusions adequately). This control is specifically important for Figure 3 and 4, whose objective is to test the impact of cell/cell contacts. But it would also be important to provide this essential control for Figure 1 and 2, as it is unclear from the images provided if MCF7 cells are forming an epithelium (and form cell/cell junctions).

Significance

The mechanosensitivity of NCT is an important question central to many aspects of cell biology. One might consider the impact of the proposed work limited, given their previous research. However, none have demonstrated that NCT is mechanosensitive in epithelial cells, making it a crucial question that needs to be addressed. Additionally, they provide a simple approach to measure NCT, which should be of interest to a broad audience.

© 2024. Published by The Company of Biologists under the terms of the Creative Commons Attribution License (https://creativecommons.org/licenses/by/4.0/).

Author response to reviewers' comments

Manuscript number: RC-2024-02344R
Corresponding author(s): Ion Andreu, Pere Roca-Cusachs

1. General Statements [optional]

We attach below a point-by-point reply to all reviewer comments. Summarizing the reviewer requests, there were three main concerns. First, both reviewers 1 and 3 mentioned that the mesenchymal versus epithelial phenotype of the two cell lines of our study was not well characterized. To address this, we have now characterized this through cadherin and actin stainings, which are shown in a new supplementary figure (supp. Fig. 1). Second, reviewer 1 suggested that the role of nuclear deformation could be further assessed in single cells through cell patterning. We have done this in new patterning experiments, which are shown in two new figures (Fig. 3 and supp. Fig. 8). Third, reviewer 1 also requested more examples of nuclear shape in 3D, which we have now provided in Fig. 1, Fig. 4, and new supplementary figures 3 and 7. Apart from these main issues, we have also addressed all other requests.

Finally, reviewer 2 had major concerns on the novelty of the work. We believe these concerns came largely from a confusion between the mechanical response of transcription factors, or of nucleocytoplasmic transport, which needed to be clarified. We have clarified this below. Regarding this, we note that the revision also included an extended discussion between the reviewers, who used the "cross-commenting" option. In this discussion, reviewers 1 and 3 largely agreed with our view. At the end of this document, we also include this discussion between reviewers, and our comments on it.

2. Point-by-point description of the revisions

We include original reviewer comments in *gray*, and our response in black.

Reviewer #1 (Evidence, reproducibility and clarity (Required)):

Summary

The nucleus is recognised as a core component of mechanotransduction with many mechano-sensitive proteins shuttling between the nucleus and cytoplasm in response to mechanical stimuli. In this work, Granero-Moya et al characterise a live florescent marker of nucleocytoplasmic transport (NCT) and how it responds to a variety of cues. This work follows on from the authors previous study (Andreu 2022) where they examined the response of passive and active NCT to mechanical signalling using a series of artificial constructs. One of these constructs (here named Sencyt) showed a differential localisation depending on substrate stiffness, accumulating in the nucleus on stiffer substrates (which the authors previously showed was due to differences in mechano-sensitivity of passive versus facilitated NCT). Here the authors use Sencyt as a tool to probe how different cues affect NCT and thus nuclear force-sensing in two different cell lines (one epithelial, one mesenchymal).

They have established a 3D image segmentation pipeline to measure both the nuclear/cytoplasmic ratio of Sencyt and 3D nuclear shape parameters. As a proof-of -principle, they show that hypoosmotic shock (which inflates the nucleus and would be expected to increase nuclear tension) and hyper-osmotic shock (which shrinks and deforms the nucleus) alter Sencyt nuclear-cytoplasmic ration as expected. They then show that inhibiting acto-myosin, which would be expected to block force transduction to the nucleus, reduces NCT, although interestingly this is without any changes to nuclear morphology. They then examine how cell density affects NCT and show that Sencyt localisation correlates only weakly with density but much more strongly with nuclear deformation (especially as measured by solidity). This is surprising considering that mechano-sensitive transcription factors such as YAP have been shown to exit the nucleus at high cell densities. Therefore, the authors directly compare Sencyt and Yap nucleo/cytoplasmic

© 2024. Published by The Company of Biologists under the terms of the Creative Commons Attribution License (https://creativecommons.org/licenses/by/4.0/).

localisation and show that Sencyt behaves differently to YAP with YAP localisation correlating strongly with cell density. This reveals an added layer of complexity in YAP regulation beyond pure changes to NCT.

Major points

The data presented throughout this work are high quality and rigorous. The controls used are appropriate (including the use of a freely diffusing mCherry to illustrate the specificity of the Sencyt probe in osmotic shock experiments - figure S2). Experiments are properly replicated and the statistical analysis is appropriate. The data are beautifully presented in figures and the manuscript is well written and very clear. Overall this is a high quality work.

We thank the reviewer for the positive assessment of the manuscript.

The discussion is careful and the conclusions are supported by the data. My only small concern is that the authors place too much emphasis on how this work is in 'multicellular systems' as opposed to their previous work in single cells (for example "Here, we demonstrate that mechanics also plays a role in multicellular systems, in response to both hypo and hyper-osmotic shocks, and to cell contractility. L212). Cell density is only controlled in figures 3 and 4 and in some of the earlier experiments, cells look quite sparse (eg Figure 2). It's also debatable how far a monolayer of cancer cells, which lack contact inhibition of growth, is a multicellular system. Furthermore, the authors don't specifically look at cell/cell adhesion or observe major differences between the epithelial or mesenchymal lines. For this reason, the authors should tone down this discussion before publication.

We agree with the reviewer that properly assessing cell-cell adhesion is important in the context of the work. To this end, we have stained for E-cadherin in both cell lines. As expected and as described previously, the results confirm that MCF7 cells do have clear cadherin-mediated cell-cell adhesions, with a cadherin staining localized specifically in cell-cell junctions. Also as expected, C26 cells show much lower cadherin expression, without a clear pattern. Further confirming this difference, MCF7 cells show clearly distinct actin organizations in their apical and basal sides, whereas C26 cells do not. Thus, we believe that the two cell models do represent a reasonable assessment of epithelial versus mesenchymal phenotypes, in a multicellular context. The data are presented in new supplementary fig. 1, and discussed in page 3 of the manuscript (first paragraph). We have also included a paragraph in the discussion to comment on the differences between cell types (page 7, 2nd paragraph).

Optional experimental suggestions: For me, the most compelling finding is that nuclear deformation has a greater correlation with NCT than cell density and that this is different from the behaviour of YAP. To cement the importance of nuclear deformation, the authors could induce deformation in single cells, for example by culture on very thin micropatterned lines and assess the localisation of Sencyt and YAP. It would also be interesting to assess the role of force transduction in this context or in different densities by removing actin, which affects NCT without inducing nuclear shape changes. These functional experiments would allow the authors to draw stronger conclusions about the role of nuclear shape and deformation but they aren't necessary for publication.

This is a very interesting suggestion. Following the reviewer's advice, we have now carried out experiments in which we have seeded cells on micropatterns of different sizes, and measured both sencyt and YAP ratios. In C26 cells, we have found as expected that increasing spreading leads to progressive nuclear deformation (as measured through nuclear solidity) and progressive increase in both sencyt and YAP ratios. Interestingly, cell spreading in MCF7 did not affect nuclear solidity, sencyt ratios, or YAP ratios. This further confirms the relationship between nuclear deformation and nucleocytoplasmic transport, and shows as well that different cell lines have different sensitivities. The lack of response of MCF7 cells is consistent with the lower sencyt response, and lower sencyt/nuclear shape correlation measured in fig. 4. It suggests that MCF7 cells may have mechanisms to shield the nucleus from deformation, something which we have reported in a different context (Kechagia et al., Nat. Mater. 2023). The new results are reported in new fig. 3, and supplementary fig. 8, and discussed in pages 5 (1st paragraph) and 6

© 2024. Published by The Company of Biologists under the terms of the Creative Commons Attribution License (https://creativecommons.org/licenses/by/4.0/).

(1st paragraph) of the manuscript results.

Minor points

- I'd like to see better examples of 3D reconstructions of nuclei (ie fig 1C but bigger) in different conditions. This is especially important in figure 3 where it would be helpful to see examples of nuclei with high or low solidity. The differences in oblateness are clear to see from the images in 3a and 3f but solidity could be better illustrated.

We have now added 3D reconstructions as requested, which illustrate the nuclear shape changes that take place. This is shown in figs. 1, 4 (which corresponds to figure 3 in the previous version of the manuscript), s3, and s7.

- Where Sencyt index is plotted, it would be clearer to add labels to at least figure 1 which indicate whether it is more cytoplasmic or nuclear.

We have done this as requested in figure 1.

Reviewer #1 (Significance (Required)):

In this work, Granero-Moya et al characterise a new tool for measuring NCT and show that it is mechanically regulated. Given the importance of NCT in mechano-transduction, this tool will be a great asset to the mechano-biology community and will likely be adopted by multiple groups in the future. The findings about the effects of cell density on NCT and differences from YAP are interesting but could be further fleshed out. This work is likely to be of greatest interest to a specialised audience working in the fields of mechano-biology and nuclear transport.

We thank the reviewer for the positive assessment.

Reviewer #2 (Evidence, reproducibility and clarity (Required)):

The study conducted by Granero Moya and colleagues describes the application of a synthetic protein which is observed to enter the nucleus in response to mechanical strains, rather than being influenced by cell density. However, the novelty of this work is minimal since the conceptual framework and the utilization of this identical or similar tool have been previously reported by the same team in earlier publications.

We respectfully disagree with the assessment of the reviewer. Please see below for a detailed response regarding novelty.

In their experiments, they employ this GFP-based sensor, referred to as Sencyt, in cells subjected to osmotic shocks. These shocks are highly stressful and impact a range of cellular processes, including stress response pathways MAPK and others; Osmoregulatory pathways; cell cycle regulations, autophagy and death pathway; ion channel regulations and others. The second findings are on cells treated with a combo of drugs affecting the actin cytoskeleton. The justification for using a combination of two specific drugs remains unclear, as the study does not adequately explain the rationale behind this choice. Additionally, there is a lack of information regarding the full range of targets these drugs affect. This raises questions about the comprehensiveness and applicability of the findings, as understanding the complete scope of the drugs' targets is crucial for interpreting the results within a minimal frame of physiological context.

The two drugs used are paranitroblebbistatin (a photostable version of blebbistatin) and Ck666. We apologize for not explaining in more detail the action of these drugs, both of which have been characterized and used extensively in the literature. Paranitroblebbistatin binds to myosin, preventing its ATPase activity and therefore impairing actomyosin contractility (https://doi.org/10.1002/anie.201403540). It acts on different myosin isoforms, including non-muscle myosin II, the main type of myosin responsible for actomyosin contractility in non-muscle

© 2024. Published by The Company of Biologists under the terms of the Creative Commons Attribution License (https://creativecommons.org/licenses/by/4.0/).

cells. CK666 binds to and inhibits arp2/3, a protein responsible for nucleating branched actin (https://doi.org/10.1016/j.chembiol.2013.03.019). This impairs lamellipodial formation and therefore cell spreading (see for instance https://doi.org/10.1371/journal.pone.0100943).

The rationale for using both drugs in combination was explained in page 4 of the manuscript. In our previous work, we determined that myosin inhibition with blebbistatin is not sufficient to inhibit nuclear mechanotransduction. Indeed, in an epithelial context, we observed that due to reduced contractility, blebbistatin-treated epithelial cells in fact spread more on their substrate. This leads to more deformed (flattened) nuclei, leading to the counterintuitive result that YAP nuclear localization increases rather than decreases. If cell spreading is impaired by interfering with branched actin nucleation, then this spreading is prevented, and the combination of drugs leads to reduced nuclear deformation, and reduced YAP nuclear localization (see supplementary fig. 7 in Kechagia et al, Nat. Mater. 2023, https://doi.org/10.1038/s41563-023-01657-3). Similar results had been published previously by the group of Clare Waterman (https://doi.org/10.1074/jbc.M115.708313).

Thus, the combination of drugs was designed to ensure that we were impairing nuclear mechanotransduction. Of course, we agree with the reviewer that all perturbations have potential side effects. Osmotic shocks will affect a range of cellular processes (as mentioned in the discussion of the manuscript), and any drug treatment can potentially have off-target effects. However, the fact that two orthogonal perturbations with different potential side effects (osmotic shocks versus actomyosin-targeting drugs) lead to the same effects in sencyt strongly suggests that the effect is mediated by mechanics, and not other factors. To reinforce this, we have now added an additional mechanical manipulation: seeding cells on micropatterned islands of different sizes. As spreading increases, cells are known to increase actomyosin contractility, and nuclear deformation (https://doi.org/10.1529/biophysj.107.116863, https://doi.org/10.1073/pnas.0235407100, https://www.nature.com/articles/ncomms1668, https://doi.org/10.1073/pnas.1902035116). As expected, nuclear solidity, sencyt ratios, and Yap ratios all increased with cell spreading. Interestingly, this occurred only for C26 and not MCF7 cells, where no changes were measured in solidity, sencyt, or YAP. The lack of response of MCF7 cells is consistent with the lower sencyt response, and lower sencyt/nuclear shape correlation measured in fig. 4. It suggests that MCF7 cells may have mechanisms to shield the nucleus from deformation, something which we have reported in a different context (Kechagia et al., Nat. Mater. 2023).

The new results are shown in figs. 3 and s8. We have also expanded the explanation of drug treatments in page 4 (3rd paragraph).

The novelty is on the specificity of this synthetic fusion protein for these manipulations and not on cell density. Yet, the reasons behind this selective response remain unexplained, potentially attributable to the unique characteristics or sensitivity thresholds of their synthetic probe. As comparison, YAP localization and this is sensitive to both inputs, but this is also already published (fig4). The focus is anyway on Sencyt for which they offer simple observations and quantifications.

The main novelty of the work lies in the characterization of the role of nucleocytoplasmic transport in mechanotransduction, in the context of multicellular systems. We and others had shown that nucleocytoplasmic transport responds to mechanical force in the context of single cells (see for instance Andreu et al. 2022 from our group, but also https://doi.org/10.1126/science.abd9776 from the Martin Beck group). However, to what extent this applies to multicellular systems was unknown. It is true that in multicellular systems, the response of YAP and other mechanosensitive transcription factors has been characterized (such as in our Elosegui-Artola 2017 paper, mostly done at the single cell level but including one figure panel on epithelial cell monolayers). The reviewer argues here and in the consultation comments with other reviewers (see below) that this demonstrated the role of nucleocytoplasmic transport in multicellular systems. However, we respectfully disagree. As also noted by reviewer 3 in the consultation, the response of YAP, and of any transcription factor, may include effects on nucleocytoplasmic transport, but will also likely include effects caused by the complex biochemical signalling pathways that regulate them.

© 2024. Published by The Company of Biologists under the terms of the Creative Commons Attribution License (https://creativecommons.org/licenses/by/4.0/).

Disentangling such effects requires a sensor that only responds to nucleocytoplasmic transport, and this is precisely what Sencyt provides.

The reviewer also states that our manuscript does not explain why sencyt responds to mechanics and not cell density. We disagree: sencyt responds to mechanics for the reasons explained in our previous work (Andreu et al., Nat. Cell Biol. 2022), and there is no reason to expect a specific response to cell density. In this regard, we don't think there are any sensitivity thresholds to detect cell density, as the probe is not designed to sense this parameter in the first place. The fact that YAP responds to both mechanics and cell density shows that the response to density cannot be merely explained by mechanics, and is rather due to signalling through other means. Of course, we agree that we do not explain the mechanism by which YAP senses cell density, but we think this lies clearly out of the scope of our manuscript.

In terms of novelty, our work also characterizes a tool to assess nucleocytoplasmic transport live in cells. We agree with the reviewer that the specific construct had been reported in our previous paper, but it had not been characterized in detail. This is done here, enabling its use by the community as a tool to measure nucleocytoplasmic transport in any context, be it related to mechanics or not.

When reviewing the figures presented, I find it challenging to detected marked differences, despite their quantitative data suggesting otherwise.

We assume here that the reviewer refers to differences in sencyt nuclear localization, that is, the sencyt index. We have now checked the example images showing changes in sencyt index, in figures 1 and 2. In figure 1, the example cells under hypo-osmotic shocks increase their sencyt index from 1.2 to 1.45 (C26). In figure 1, the example cells under hyper-osmotic shocks decrease their sencyt index from 0.9 to 0.3 (MCF7) and from 1.4 to 0.5 (C26). In figure 2, the example cells increase their sencyt index upon drug washout from 0.2 to 1.4 (MCF7) and from 0 to 0.9 (C26). Of course, these individual values don't reflect exactly average values, but they do reflect the reported average trends and their magnitudes faithfully. Here we note that even though sencyt changes with the different treatments, it is always more nuclear than cytosolic (sencyt index >0, as it has an NLS). Thus, to the naked eye, sencyt always seems to show a "bright" nucleus, and it is hard to intuitively see changes in its localization. Further, we also note that osmotic shocks lead to overall changes in fluorescence levels due to volume changes (as GFP molecules get diluted or concentrated in hypo or hyper osmotic shocks, respectively). This does not affect ratiometric quantifications as assessed with our mcherry control, but means that changes in ratios are hard to see by eye. To help in this visualization, we have now changed the images from green to grayscale, which is better perceived by the human eye. We have also specified the issue of fluorescence intensity changes in the legend of the figure.

In addition to this, we have seen that there is indeed a case in which examples were not following average trends. In the case of hypo-osmotic shocks in figure 1, example MCF7 cells were barely changing their sencyt index with treatment. We apologize for choosing this non-representative image for the figure, we have now changed the figure to show more representative cells.

Furthermore, the study attempts to correlate the behavior of Sencyt with the nuclear geometric parameter of solidity, a connection that seems to lack a clear basis in cell biology and could potentially lead to misconceptions.

Mechanical effects on nucleocytoplasmic transport are mediated by mechanical tension application to nuclear pores, which are embedded in the nuclear membrane (nuclear envelope). Whereas nuclear envelope tension is very challenging to measure directly, it can be indirectly related to nuclear shape. Indeed, a tense membrane will tend to even out membrane irregularities and appear rounded, whereas a membrane under low tension will tend to show wrinkles. Nuclear solidity is a geometric parameter that compares actual nuclear volume to the volume of the convex hull (intuitively, the volume of the smallest wrinkle-free object containing all of the nucleus). Thus, it is the geometric parameter that best reflects the presence of wrinkles, folds or irregularities, and as such the one that should best correlate to membrane tension. Of course, this correlation is not perfect, and there could be many situations in which

© 2024. Published by The Company of Biologists under the terms of the Creative Commons Attribution License (https://creativecommons.org/licenses/by/4.0/).

changes in membrane tension may not directly affect nuclear solidity. But we do believe that solidity is the geometrical parameter that should best reflect membrane tension, and this is why we focus on it. Consistent with our hypothesis, solidity is the geometrical parameter that best correlates with sencyt. To further clarify this, we now explain this rationale in detail in page 4 of the manuscript (1st paragraph).

Reviewer #2 (Significance (Required)):

In sum, I think the MS is of interest for a very specialistic audience. There are no clear interpretations. The work is done in one or two cellular model systems in vitro; and the general significance of these observations is of very limited impact and no novelty.

We strongly disagree. The study is done on two cellular models, one with epithelial and the other with mesenchymal phenotype, and thus highly relevant for multicellular systems. Following suggestions by reviewers 1 and 2, we have now characterized the epithelial/mesenchymal behaviour of the cell types in detail (see supp. fig. 1). The results are novel in that they demonstrate the role of nucleocytoplasmic transport in multicellular systems, something which as argued above had not been done before. The difference with YAP, and the disentanglement between transport and signalling, is also novel. Finally, we believe the manuscript will be impactful because of this novelty, but also because of the availability of sencyt as a tool for the community. In fact, since placing this manuscript in biorxiv, we have received many requests (directly and through addgene) to share sencyt, which is currently being used in several labs across the world.

Reviewer #3 (Evidence, reproducibility and clarity (Required)):

In this very well-written manuscript, Pere Roca-Cusachs and colleagues investigated the response of nucleocytoplasmic transport (NCT) to mechanical stress and tested whether this response is similar in epithelial and mesenchymal cells using a combination of quantitative approaches. This study builds upon their earlier findings, which elegantly demonstrated that NCT is sensitive to mechanical forces transmitted to the nuclear membrane. Using a similar approach to their recent work, they quantitatively analyzed NCT and compared the two cell types using various treatments that impact nuclear membrane tension. The study is straightforward and experimentally sound, with an adequate number of replicates and independent experiments. While one might consider the limitations given their previous work, none have demonstrated that NCT is mechanosensitive in epithelial cells. Additionally, they provide a simple approach to measure NCT, which should be of interest in the field. However, it is unclear how the authors defined the epithelial phenotype in this work and whether they solely based this characterization on the tissue/cell's origin. Epithelia can be defined ultrastructurally with reference to their apico- basal polarity and specific cell-cell junctions (Alberts et al., 1994; Davies and Garrods, 1997). Changing cell density should affect cell/cell adhesion, but the authors provide no evidence that the cells tested in the study are attached to their neighbors on all sides and form an epithelium. While I recognize that the objective of this study is not to mimic the in vivo behavior of epithelial tissue, the authors should at least ensure that cells form a monolayer by quantitatively assessing cell-cell junctions (or they should adjust their conclusions adequately). This control is specifically important for Figure 3 and 4, whose objective is to test the impact of cell/cell contacts. But it would also be important to provide this essential control for Figure 1 and 2, as it is unclear from the images provided if MCF7 cells are forming an epithelium (and form cell/cell junctions).

We thank the reviewer for the positive assessment of our work. We fully agree with the reviewer that properly assessing cell-cell adhesion is important in the context of the work. To this end, we have stained for E-cadherin in both cell lines. As expected and as described previously, the results confirm that MCF7 cells do have clear cadherin-mediated cell-cell adhesions, with a cadherin staining localized specifically in cell-cell junctions. Also as expected, C26 cells show much lower cadherin expression, without a clear pattern. Further confirming this difference, MCF7 cells (but not C26 cells) show a clear apico-basal polarization, with distinct actin organizations in their apical and basal sides. Thus, we believe that the two cell models do represent a reasonable

© 2024. Published by The Company of Biologists under the terms of the Creative Commons Attribution License (https://creativecommons.org/licenses/by/4.0/).

assessment of epithelial versus mesenchymal phenotypes, in a multicellular context. The data are presented in new supplementary fig. 1. We have also included a paragraph in the discussion to comment on the differences between cell types (page 7, 2nd paragraph).

Reviewer #3 (Significance (Required)):

The mechanosensitivity of NCT is an important question central to many aspects of cell biology. One might consider the impact of the proposed work limited, given their previous research. However, none have demonstrated that NCT is mechanosensitive in epithelial cells, making it a crucial question that needs to be addressed. Additionally, they provide a simple approach to measure NCT, which should be of interest to a broad audience.

We thank again the reviewer for this positive assessment.

Referees cross-commenting

Here comments from all 3 reviewers are reported

Reviewer 1:

I disagree with R2's comment that there is 'no novelty' here. Although this work is going to be of greater interest to a specialised rather than general audience, it characterises in depth a simple tool to measure NCT which will be useful for mechanobiology field. Also, using 'two cellular model systems in vitro' is very standard in the field when assessing subcellular processes like NCT. Using this approach in vivo would be very interesting but challenging and would be an entirely different study.

I agree with R2's comments that the authors should better justify their combination of two actin inhibitors and R3s point on better assessing cell/cell junctions.

We thank the reviewer for these comments. Both issues have been addressed, as described in the response to reviewers above.

Reviewer 2

About Reviewer 3's comments, I believe it's a stretch to highlight the strength and novelty based on "NCT's mechanosensitivity in epithelial cells has not been demonstrated,". There are thousands of papers on the Hippo pathway, that is known to be mechanosensitive, on the regulation of YAP, that enters in the nucleus in Hippo inhibited conditions and exits to the cytoplasm in Hippo induced cells, including downstream of mechanical signals. The phenomenon of nuclear-cytoplasmic shuttling being a common event from neurons to endothelial and multiple types of epithelial, immune, and fibroblast cells is already established through NCT of this and other endogenous proteins. This is simply an accepted fact. Then, The Nature cell Biology 2022 was offering a very general claim. No warning that conclusions could have been cell type specific. In the Artola 2017 Cell paper they also showed NCT in mammary epithelial cells. We should definitively conclude that NCT's mechanosensitivity in epithelial cells has been well demonstrated.

We disagree with this assessment, for the same reasons also exposed by reviewer 3 below. Previous work on YAP and other transcription factors cannot be seen as a demonstration of the role of nucleocytoplasmic transport per se. The localization of any transcription factor is highly regulated by complex signalling pathways, and can be affected by many factors. One of them is nucleocytoplasmic transport, but signalling events (for instance through phosphorylation) could change localization by promoting binding to cytosolic or nuclear binding partners, by promoting protein degradation, by masking nuclear localization signals, and others. To isolate the role of nucleocytoplasmic transport, a probe sensitive only to this factor should be designed. This is exactly what sencyt provides. In fact, this has allowed us to answer an important open question: is the sensitivity of YAP to cell density mediated by mechanics and nucleocytoplasmic transport,

© 2024. Published by The Company of Biologists under the terms of the Creative Commons Attribution License (https://creativecommons.org/licenses/by/4.0/).

or is it mediated by some other factor? Our results suggest that some other factor, likely mediated by the Hippo pathway and not necessarily mechanotransduction, explains this sensing of cell density. This is a novel finding, which was not provided in either our Elosegui-Artola 2017 paper or our Andreu 2022 paper.

About Reviewer 1: I find it challenging to grasp the point made in the comment. On novelty, in their previous study in NBC 2022 Syncet was already shown to undergo NCT. The reviewer states that the study presents "a simple tool to measure nuclear-cytoplasmic transport (NCT) beneficial for the mechanobiology field, and evidence that this demonstrates a novel layer of regulation in hippo signaling (also because this is observational and not a mechanistic study). The tool in question is far from simple. Its application requires transfection into cell cultures, conducting live imaging, etc. If one aims to measure NCT of endogenous proteins, straightforward immunofluorescence or live imaging of endogenous proteins (like GFP-tagged YAP, Twist, Smads, etc.) using the same experimental setup should suffice to demonstrate relevance, without necessitating any additional experiments. What then, is the unique benefit of this proposed tool? Given it's an artificial construct combining NLS-GFP with a bacterial protein, questions arise about the effects of the forced nuclear localization signal (NLS) or the bacterial component. It is an empirical artificial construct and there is no mechanism to explain its behavior. The comparison of Syncet with YAP seems to me questionable and of limited utility.

As also noted by reviewer 3 below, the use of genetically encoded fluorescent sensors that require transfection is by now absolutely standard in biology, and cannot be considered to be “far from simple”. And as stated above, imaging of endogenous transcription factors (which also requires transfection if it is done live) does not isolate the role of nucleocytoplasmic transport. We also disagree that “there is no mechanism to explain its behaviour”. Sencyt was developed in our previous andreu et al 2022 paper, where the mechanism is explained in detail.

It's unsurprising that an artificial construct only mirrors some aspects of what is considered a genuine mechanosensitive protein. The utility of a synthetic tool lies in its ability to replicate actual phenomena, not in what it fails to do. In comparison to their NBC 2022 study, this manuscript focuses on what their reporter fails to detect.

We disagree that a synthetic tool is only useful if it replicates the behaviour of endogenous proteins. A synthetic tool, precisely due to its engineered, artificial nature, can be made to respond only to specific factors (in this case, nucleocytoplasmic transport). This can then be used to disentangle the role of such specific factors, as done here.

The osmotic shock was the assay in their 2017 Cell paper. Here they demonstrate that a combination of Blebbistatin+CK (an unclear choice of drugs) is ineffective, as is cell density. Are there other specific peculiarities associated with this construct?

Here, we note that our osmotic shock experiments in our 2017 paper were done for YAP (not nucleocytoplasmic transport in general). Regarding the choice of drugs, please refer to our answer to the reviewer comments above for a full explanation. Also, we want to clarify that this combination is not ineffective, as it leads to clear changes in sencyt.

My other concern is on the minor quantitative changes reported, which seem inconsistent with the provided representative images, where significant differences are difficult to appreciate. For instance, the claim that the transfected sensor differs from an endogenous NCT protein, YAP, after cell density treatment, is hard to detect in their images. In Figure 4, comparing YAP and Syncet in C26 cells, YAP appears uniformly nuclear at high cell density, potentially more nuclear than the synthetic sensor, which is not coherent with their claim.

Regarding the concern of the minor changes seen in images, please refer to our full response to the reviewer comments above. Regarding the comparison between sencyt and YAP, we want to clarify that in our manuscript we do not compare the absolute values of nuclear localization between YAP and sencyt. As the reviewer notes, these are two different proteins, so which one is more nuclear does not really provide useful information. So whether YAP is more or less nuclear than sencyt is unrelated to (not incoherent with) our claim. What we state in figure 4 is that YAP responds to cell density, whereas sencyt does not. This is clear from the quantifications and also

© 2024. Published by The Company of Biologists under the terms of the Creative Commons Attribution License (https://creativecommons.org/licenses/by/4.0/).

from the images.

From the Hippo perspective, there is really an unusual amount of nuclear YAP left in their cells. This should be almost completely cytoplasmic from prior contact inhibition studies in the Hippo field. Syncet could be simply less sensitive than YAP in these borderline conditions. Although there's a more noticeable cytoplasmic noise in dense cells with YAP compared to Syncet, this could be attributed to several factors, including differences in protein degradation rates, which I suspect to be quicker for a synthetic protein. From a technical perspective it is complex to get strong conclusions after comparing something so unrelated with each other. One is a live GFP detection and the other is a staining by immunofluorescence. the nature of the background is also different and so conclusions from comparisons between unrelated systems is not justified.

In conditions of high density, average YAP ratios are close to one (zero in logarithmic scale, as reported in the figures) for MCF10A cells, so there is no nuclear localization. This is similar to what we and others have previously reported in similar conditions (Elosegui Artola et al 2017, Kechagia et al. 2023, for example). In C26 cells, YAP levels at high density are a bit higher. This is likely due to their mesenchymal nature, and therefore diminished cell-cell contact inhibition (as assessed in detail in this revision). This in fact further suggests that the response of YAP to cell-cell contacts is different from a mere mechanical factor, supporting our hypothesis. Regarding the issue of noise, background noise is removed from quantifications, and potential noise coming from non-specificities or autofluorescence is also cancelled by the fact that we compute fluorescence ratios between nucleus and cytoplasm (and not absolute values). Thus, we don't think noise is an issue. Further, we note again that we do not directly compare values between sencyt and yap.

This suggests caution on what is heralded as the main claim here put forward.

Reviewer 1:
I do have some sympathy with R2s comments in the consultation. I agree that showing that NCT is mechanosensitive in an epithelium is not new. I also agree that sometimes it is difficult to see the quantitative differences by eye. This second point could be addressed by including more details of the segmentation and analysis in the supplemental material (along with some example images).

We thank the reviewer for the suggestions. Regarding the novelty, please see above for a detailed discussion, and also the comments of reviewer 3 below (previous work studied not NCT but transcription factors, affected by many parameters). Regarding quantitative differences, we have now addressed this issue by showing images in grayscale rather than green, and also by replacing one example cell in figure 1 which indeed did not reflect the average measured trends. We now also show examples of 3D rendered images of the nuclei in different conditions. We have also gone through the methods and clarified in detail how ratios are calculated, the segmentation procedure is also explained in detail.

Regarding novelty, I would be interested to know if R2 thinks that there are experiments that the authors could do to improve the work. Or do they need to simply tone down their claims? It's perfectly acceptable to publish a well characterised tool with a series of observations and it's beneficial to the community to do so.

Reviewer 3

Thanks to Reviewers #1 and #2 for using this consultation option; I truly appreciate their feedback on my comments and find it extremely valuable. I agree with Reviewer #1 that the method proposed here is relatively simple. Transfecting cells and conducting live fluorescent imaging can hardly be considered difficult. I believe the construct used/designed by the authors is the main advantage as it provides a specific way to quantitatively assess NCT and not limit the analysis to a single nuclear protein (such as YAP). Reviewer #2 suggests using immunofluorescence staining of YAP or live imaging of fusion fluorescent protein (following transfection) to analyze NCT, but this approach would yield a readout not only based on NCT but

© 2024. Published by The Company of Biologists under the terms of the Creative Commons Attribution License (https://creativecommons.org/licenses/by/4.0/).

also on the many other interacting partners/mechanisms that regulate the candidate localization, resulting in an unspecific readout (and similar transfection/live imaging set-up).

We thank the reviewer for this comment, we fully agree and have elaborated on this in our responses above.

Regarding the impact of the study, I agree that it is certainly not as impactful as previous publications on this topic. Although I find reviewer#2 argument on Yap irrelevant, as YAP is not the main focus of this paper. Some experiments have been done with cells of epithelial origin, but NCT mechanosensitivity has not been clearly tested in epithelial monolayer, which is the main claim of the proposed study here. The 2017 Cell paper focused on YAP transport into the nucleus (and not NCT in general) and they showed a correlation between YAP nuclear localization and traction force in MCF10A. I am not sure if one would say that "NCT mechanosensitivity has been well demonstrated in epithelial cells" based on this single panel. The impact of the proposed study is certainly not outstanding but offering a thorough analysis in epithelial cells (as monolayers and not as individual cells) and presenting a well-defined experimental approach should be of interest in the field. I agree with comments from reviewer#2 that some reported effects in graph are unclear on main images. More experimental details should hopefully clarify this aspect.

We fully agree with the reviewer. Regarding quantitative differences, we have now addressed this issue by showing images in grayscale rather than green, and also by replacing one example cell in figure 1 which indeed did not reflect the average measured trends.

Original submission

Original submission

<u>First decision letter</u>

MS ID#: JOCES/2024/262363

MS TITLE: Nucleocytoplasmic transport senses mechanics independently of cell density in cell monolayers.

AUTHORS: Ignasi Granero-Moya, Valeria Venturini, Guillaume Belthier, Bart Groenen, Marc Molina, Miguel Gonzalez, Xavier Trepat, Jacco van Rheenen, Ion Andreu, and Pere Roca-Cusachs
ARTICLE TYPE: Research Article

We have now reached a decision on the above manuscript.

To see the reviewers' reports and a copy of this decision letter, please go to: https://submit-jcs.biologists.org and click on the 'Manuscripts with Decisions' queue in the Author Area. (Corresponding author only has access to reviews.)

As you will see, the reviewers gave favourable reports but reviewers 1 and 2 made minor suggestions that I would like for you to consider and respond to; reviewer 3 is satisfied. These issues are in the plotting style and abstract text and so do not involve new experiments. I hope that you will be able to carry these out because I would like to be able to accept your paper. Please be clear in your response so that I can assess the revised manuscript directly.

Please ensure that you clearly highlight all changes made in the revised manuscript. Please avoid using 'Tracked changes' in Word files as these are lost in PDF conversion.

I should be grateful if you would also provide a point-by-point response detailing how you have dealt with the points raised by the reviewers in the 'Response to Reviewers' box. Please attend to

© 2024. Published by The Company of Biologists under the terms of the Creative Commons Attribution License (https://creativecommons.org/licenses/by/4.0/).

all of the reviewers' comments. If you do not agree with any of their criticisms or suggestions please explain clearly why this is so.

Reviewer 1

Advance summary and potential significance to field

As stated in my review for Review Commons, this is a high quality work that characterises a tool for monitoring NCT that is likely to be of great interest to the cell biology community. The authors have used this tool to study NCT in cell monolayers and importantly, reveal key differences between generic NCT and the behaviour of the mechano-sensitive transcription factor YAP in response to cell density.

In the revision, the authors convincingly address all the reviewers' points.
They have added clarity to the figures by changing the images to greyscale and by showing more nuclei 3D reconstructions. They have also added new data showing cell/cell junctions in MCF7 cells and examining the effect of cell spreading on NCT using micropatterns. The revision has improved this work and I recommend publication in J Cell Sci.

Comments for the author

Minor comments:
- Some of the graphs (eg figure 5 -previously 4 & Supp figure 6) have been changed from plotting points to lines. The previous version (points) was better as it gives information on the sampling frequency and the authors should revert to this original format before publication.

Reviewer 2

Advance summary and potential significance to field

I think this is an interesting paper that should be published. The data extend their prior conclusion on the synthetic sensor Sencyt previously reported by the same authors in nature cell biology. The new data are convincing, advancing over prior work and supporting the interest of this reagent, and there is no need from this reviewer to request additional experiments. I am however only hesitant on the conclusion that I see in the abstract, that is in fact better and more carefully/conservatively discussed in the text. I would not stretch that:
[In contrast and in opposition to YAP, NCT is not affected by cell density, showing that the response of YAP to both mechanics and cell-cell contacts operates through distinct mechanisms. Our results demonstrate the generality of the mechanical regulation of NCT].
Sencyt was generated by iterative optimization of NLS-GFP with repeats of the 7kDa bacterial protein PrA.
There may be several explanations on why Sencyt overlaps with some but not all YAP responses. Then, the paper describes no mechanisms and so the author should abstain from claiming differences in mechanisms.
NCT of endogenous YAP is the generality of mechanical regulation in real cells/contexts. How and compared to what (natural or synthetic) should be the focus of future work.
I congratulate authors for their work but they should delete that statement, or say something like: [Differently from YAP, NCT is not affected by cell density suggesting additional regulatory layers and crosstalks in the regulation of endogenous mechanosensory proteins, and thus the utility of Syncet as tool for fine and specific experimental control of NCT in mechanoresponses].

Comments for the author

see above

Reviewer 3

© 2024. Published by The Company of Biologists under the terms of the Creative Commons Attribution License (https://creativecommons.org/licenses/by/4.0/).

Advance summary and potential significance to field

Building upon their previous findings, which showed that nucleocytoplasmic transport (NCT) is sensitive to mechanical forces, Pere Rochas-Cusachs and colleagues compared NCT in epithelial and mesenchymal cells using a combination of quantitative approaches. The authors found that NCT correlates with the degree of nuclear deformation in epithelial cells and is not affected by cell density. The conclusions seem appropriate, and the study is experimentally solid and likely to attract interest in the field.

Comments for the author

As Reviewer 3 of the previous review cycle in Review Commons, I am satisfied with the authors' response and recommend this revised work for publication in JCS.

First revision

Author response to reviewers' comments

Response to reviewer comments

Reviewer 1 Advance Summary and Potential Significance to Field:

As stated in my review for Review Commons, this is a high quality work that characterises a tool for monitoring NCT that is likely to be of great interest to the cell biology community. The authors have used this tool to study NCT in cell monolayers and importantly, reveal key differences between generic NCT and the behaviour of the mechano-sensitive transcription factor YAP in response to cell density.

In the revision, the authors convincingly address all the reviewers' points. They have added clarity to the figures by changing the images to greyscale and by showing more nuclei 3D reconstructions. They have also added new data showing cell/cell junctions in MCF7 cells and examining the effect of cell spreading on NCT using micropatterns. The revision has improved this work and I recommend publication in J Cell Sci.

We thank the reviewer for the positive assessment.

Reviewer 1 Comments for the Author:

Minor comments:

- Some of the graphs (eg figure 5 -previously 4 & Supp figure 6) have been changed from plotting points to lines. The previous version (points) was better as it gives information on the sampling frequency and the authors should revert to this original format before publication.

We have reverted the graphs to the original format as requested.

*****

Reviewer 2 Advance Summary and Potential Significance to Field:

I think this is an interesting paper that should be published. The data extend their prior conclusion on the synthetic sensor Sencyt previously reported by the same authors in nature cell biology. The new data are convincing, advancing over prior work and supporting the interest of this reagent, and there is no need from this reviewer to request additional experiments.

We thank the reviewer for the positive assessment of the manuscript.

© 2024. Published by The Company of Biologists under the terms of the Creative Commons Attribution License (https://creativecommons.org/licenses/by/4.0/).

I am however only hesitant on the conclusion that I see in the abstract, that is in fact better and more carefully/conservatively discussed in the text. I would not stretch that:

[In contrast and in opposition to YAP, NCT is not affected by cell density, showing that the response of YAP to both mechanics and cell-cell contacts operates through distinct mechanisms. Our results demonstrate the generality of the mechanical regulation of NCT].

Sencyt was generated by iterative optimization of NLS-GFP with repeats of the 7kDa bacterial protein PrA. There may be several explanations on why Sencyt overlaps with some but not all YAP responses. Then, the paper describes no mechanisms and so the author should abstain from claiming differences in mechanisms. NCT of endogenous YAP is the generality of mechanical regulation in real cells/contexts. How and compared to what (natural or synthetic) should be the focus of future work. I congratulate authors for their work but they should delete that statement, or say something like:

[Differently from YAP, NCT is not affected by cell density suggesting additional regulatory layers and crosstalks in the regulation of endogenous mechanosensory proteins, and thus the utility of Syncet as tool for fine and specific experimental control of NCT in mechanoresponses].

We agree with the reviewer that our work does not demonstrate the mechanisms by which YAP and Sencyt respond differently. As suggested, we have removed the word “mechanism” from the abstract, and we have rewritten the section as:

In contrast, YAP but NCT is sensitive to cell density, showing that YAP response to cell-cell contacts is not via a mere mechanical effect of NCT. Our results demonstrate the generality of the mechanical regulation of NCT.

Reviewer 2 Comments for the Author:

see above

*****

Reviewer 3 Advance Summary and Potential Significance to Field:

Building upon their previous findings, which showed that nucleocytoplasmic transport (NCT) is sensitive to mechanical forces, Pere Roca-Cusachs and colleagues compared NCT in epithelial and mesenchymal cells using a combination of quantitative approaches. The authors found that NCT correlates with the degree of nuclear deformation in epithelial cells and is not affected by cell density. The conclusions seem appropriate, and the study is experimentally solid and likely to attract interest in the field.

Reviewer 3 Comments for the Author:

As Reviewer 3 of the previous review cycle in Review Commons, I am satisfied with the authors' response and recommend this revised work for publication in JCS.

We thank the reviewer for the positive assessment of the manuscript.

Second decision letter

MS ID#: JOCES/2024/262363

MS TITLE: Nucleocytoplasmic transport senses mechanics independently of cell density in cell monolayers.

© 2024. Published by The Company of Biologists under the terms of the Creative Commons Attribution License (https://creativecommons.org/licenses/by/4.0/).

AUTHORS: Ignasi Granero-Moya, Valeria Venturini, Guillaume Belthier, Bart Groenen, Marc Molina-Jordan, Miguel Gonzalez-Martin, Xavier Trepat, Jacco van Rheenen, Ion Andreu, and Pere Roca-Cusachs
ARTICLE TYPE: Research Article

Thank you for sending your manuscript to Journal of Cell Science through Review Commons.

I am happy to tell you that your manuscript has been accepted for publication in Journal of Cell Science, pending standard publication integrity checks.

© 2024. Published by The Company of Biologists under the terms of the Creative Commons Attribution License (https://creativecommons.org/licenses/by/4.0/).
